# Long-Term Proton Pump Inhibitor–Acid Suppressive Treatment Can Cause Vitamin B_12_ Deficiency in Zollinger–Ellison Syndrome (ZES) Patients

**DOI:** 10.3390/ijms25137286

**Published:** 2024-07-02

**Authors:** Tetsuhide Ito, Irene Ramos-Alvarez, Robert T. Jensen

**Affiliations:** 1 Neuroendocrine Tumor Centra, Fukuoka Sanno Hospital, International University of Health and Welfare, 3-6-45 Momochihama, Sawara-Ku, Fukuoka 814-0001, Japan; itopapa@kouhoukai.or.jp; 2 Digestive Diseases Branch, NIDDK, NIH, Bethesda, MD 20892-1804, USA; irene.ramosalvarez@nih.gov

**Keywords:** Zollinger–Ellison syndrome, PPI, vitamin B_12_ deficiency, acid hypersecretion, neuroendocrine tumor, gastrinomas, total homocysteine, methylmalonic acid

## Abstract

Whether the long-term treatment of patients with proton pump inhibitors (PPIs) with different diseases [GERD, Zollinger–Ellison syndrome (ZES), etc.] can result in vitamin B_12_ (VB_12_) deficiency is controversial. In this study, in 175 patients undergoing long-term ZES treatment with anti-acid therapies, drug-induced control acid secretory rates were correlated with the presence/absence of VB_12_ deficiency, determined by assessing serum VB_12_ levels, measurements of VB_12_ body stores (blood methylmalonic acid (MMA) and total homocysteine[tHYC]), and other features of ZES. After a mean of 10.2 yrs. of any acid treatment (5.6 yrs. with PPIs), 21% had VB_12_ deficiency with significantly lower serum and body VB_12_ levels (*p* < 0.0001). The presence of VB_12_ deficiency did not correlate with any feature of ZES but was associated with a 12-fold lower acid control rate, a 2-fold higher acid control pH (6.4 vs. 3.7), and acid control secretory rates below those required for the activation of pepsin (pH > 3.5). Over a 5-yr period, the patients with VB_12_ deficiency had a higher rate of achlorhydria (73% vs. 24%) and a lower rate of normal acid secretion (0% vs. 49%). In conclusion, in ZES patients, chronic long-term PPI treatment results in marked acid hyposecretion, resulting in decreased serum VB_12_ levels and decreased VB_12_-body stores, which can result in VB_12_ deficiency.

## 1. Introduction

The pharmacological control of gastric acid secretion by increasingly potent classes of gastric acid antisecretory drugs has been one of the most successful pharmaceutical accomplishments over the last 50 years. This progression started with the development of histamine H_2_-receptor antagonists in the 1970s (metiamide, followed by cimetidine, ranitidine, famotidine, nizatidine, etc.), followed by the introduction of inhibitors of the gastric H+K+ ATPase (proton pump inhibitors) (PPIs) in the 1980s (omeprazole, followed by lansoprazole, esomeprazole, pantoprazole, and rabeprazole) [1,2,3] and, very recently, by the introduction of gastric potassium-competitive acid blockers such as vonoprazan [4,5,6,7]. PPIs have been one of the best-selling drugs in the US as well as worldwide for several years [1,2,3], and in 2019, it was the eighth most commonly prescribed medication in the US, with >52 million prescriptions [8], resulting in 7–15% of patients using these drugs at some point [9,10,11]. With the recent availability of PPIs without prescriptions, because of their availability as over-the-counter drugs, the use of PPIs is increasing even further [12]. PPIs have overwhelmingly proven to be safe and effective drugs and are the mainstay for the treatment of gastroesophageal reflux disease (GERD) and peptic ulcer disease, for which they have been approved [1,2,13]. However, PPIs are increasingly being used with less clear indications [12,14,15] and, in fact, in a recent study [16], in nearly two-thirds of the patients, they were used with no clear indications.

This increased use of PPIs, coupled with the fact there is increased long-term use and even potential lifetime treatment with PPIs [17], especially for patients with chronic, advanced GERD, because the symptoms return quickly when the PPI is stopped, has led to increasing concerns about the long-term safety of these drugs [9,10,18,19,20,21,22,23,24,25,26,27,28,29,30,31,32]. These safety concerns involve not only potential side effects of the PPI’s action but also the development of long-term hypergastrinemia, which invariably develops with their continued use [10,33,34,35,36,37]. These safety concerns have also been heightened by numerous reports, primarily from epidemiological or observational studies of potential serious side effects linked to PPIs [9,10,19,22,23,24,25,26,27,31,38], including bone fractures [18,30,39,40,41,42,43,44,45], chronic renal disease [46,47,48,49,50,51], malabsorption of nutrients [18,52,53,54,55,56], infections [57,58,59,60,61], increased cancer risk [33,34,36,62,63], increased mortality [64,65,66], increased drug–drug interactions with other therapeutic agents [38,67,68], dementia [69,70,71,72], and an increase in other CNS effects [69,73,74,75,76]. Although causality has not been proven by these studies and randomized control studies do not show an increased incidence of side effects [9,38], these concerns persist.

One of the safety issues that remains contentious and unresolved is in regard to the effect of chronic long-term treatment with PPIs on the absorption of the essential nutrient vitamin B_12_ (VB_12_) and whether it lowers serum vitamin B_12_ levels/body stores to the extent that vitamin B_12_ deficiency can develop, and if it this does occur, through which mechanism do PPIs cause this [18,52,53,54,77,78,79]? This controversy exists not due to a lack of research on this topic but because of the differences in the results of the studies that have been performed. Many of these studies have been performed in patients chronically taking PPIs for GERD, some of which support the conclusion that there is a long-term treatment effect of PPIs, which can result in a decrease in serum VB_12_ levels/body stores and, thus, the development of VB_12_ deficiency [54,78,80,81,82,83,84,85,86,87,88,89,90,91,92,93,94]. However, numerous other studies do not support this conclusion and show no effect of PPIs on VB_12_ stores or the development of VB_12_ due to PPI usage [79,95,96,97,98,99]. One of the problems with these studies is that except for two studies on patients with Zollinger–Ellison syndrome (ZES) [54,79], none of the other studies found a correlation between the effect of the PPI on acid secretory rate and its effect on VB_12_ body stores; so, there was no clear relationship between the direct effect of PPI reduction on acid secretion and the effect on VB_12_ body stores, and thus, they did not provide any insight into the mechanism(s) involved regarding the observed effects of PPIs. The two studies on VB_12_ status with chronic PPI treatment in ZES patients [54,79] were performed to address this question. They were performed in ZES patients because of their need for life-long anti-acid treatment, which was shown by the fact that prior to the availability of potent acid antisecretory drugs, these patients died from their massive acid hypersecretion unless successful surgical treatment was performed (vagotomies, gastric resection, and total gastrectomies). Moreover, because only 25% of these patients are cured surgically [100,101,102], 75% require life-long treatment with PPIs with regular measurements of drug-induced acid secretory rates to adjust the drug dose [103,104,105] because of the continued acid hypersecretion secondary to the chronic hypergastrinemia caused by the continued ectopic release of gastrin from the gastrinomas [100,101,102]. In both of these ZES studies [54,79], it was found that there was a decrease in serum VB_12_ with long-term PPI treatment, but in one case [54], it was shown that the change in VB_12_ levels was due to the PPI-induced hypo-/achlorhydria; however, no study was performed on VB_12_ body stores to determine whether VB_12_ deficiency actually developed or examine the relationship between any changes in VB_12_ levels and the continued PPI treatment or the possible mechanisms involved. In the second study [79], it was concluded that the decrease induced by PPIs in the acid secretion of the ZES patients could not account for the change in serum VB_12_ or VB_12_ deficiency, which developed in some patients. Despite these unclear results in these two ZES studies, there are several reasons that the potential study of ZES patients could still provide one of the best opportunities to resolve the issue of whether prolonged PPI use in humans affects VB_12_ stores, resulting in VB_12_ deficiency, and to provide insights into the possible mechanisms involved. ZES patients are among the very few patient groups that have regular acid control assessments at all yearly admissions [103,104,105,106,107,108,109,110,111], when they are required to undergo an assessment of the adequacy of the acid secretory control and adjustment of the PPI dose to have secretory control to acceptable levels [103,104,105,106,107,108,109,110,111]. The result of this regular monitoring of acid secretion control allows the acid secretory status induced by the PPI to be continuously determined, which can then be correlated with other factors such as changes in VB_12_ body stores. This can lead to the potential assessment of effects of PPI-induced acid suppression on VB_12_ body stores or other PPI-induced effects. Therefore, in the current study, we investigated in detail the relationships of the PPI-induced acid secretory status in ZES patients with changes in VB_12_ body stores, including effects on both VB_12_ serum levels and VB_12_ body status by assessing blood methyl malonic acid levels and total homocysteine levels, which are well-established markers of body VB_12_ stores and VB_12_ deficiency [112,113,114,115,116,117,118,119,120,121,122]. In addition to defining the mechanism(s) of any changes in VB_12_ stores, we also investigated in detail any other factors that might contribute to these changes in VB_12_ levels, including clinical, tumoral, or laboratory features of ZES. By comparing these changes in patients treated with PPIs to the changes in acid/VB_12_ levels seen in patients on H_2_Rs, as well as the presence or absence of VB_12_ deficiency, we were able to provide evidence that the longstanding, chronic PPI treatment in our patients results in a decrease in serum VB_12_ levels and VB_12_ body stores, which can result in VB_12_ deficiency; this is mediated by the effect of PPI-induced hypo-/achlorhydria, which can cause VB_12_ mal-digestion/malabsorption, supporting the conclusion that chronic PPI treatment can result in VB_12_ deficiency under the conditions of this study.

## 2. Results

### 2.1. Patient Characteristics [Clinical, Laboratory, Tumoral, Acid Treatment Duration, Drug Type/Dosage, and Result]

One hundred and seventy-five consecutive patients with ZES were included in this study (Table 1). The clinical, laboratory, and tumoral features of the 175 patients are shown in Table 1 and Table 2. The patient’s clinical characteristics were similar to those in most other large series of ZES patients, which were predominantly male and Caucasian and mainly had the sporadic form (74%) of ZES [100,123,124,125,126,127,128,129,130,131,132]. Similarly, the patients resembled those in other series, with a 6-year delay in diagnosis, and at the time of this study, they were middle-aged (age 54 yrs.), with an average of 4 years after their initial evaluation at the NIH, and presented with clinical features usually described in other large series, most prominently including pain, diarrhea, and gastroesophageal reflux disease (GERD) [100,123,124,126,131,133,134,135,136,137,138]. The patients also had a long follow-up at the NIH, almost 20 years since ZES onset and over 10 years since their first admission at the NIH (Table 1). Similarly, the laboratory features with marked hyperchlorhydria/hypergastrinemia characteristic of ZES patients were present with both basal and maximal acid outputs that were markedly increased both in patients with and without previous gastric acid-reducing surgery and marked fasting hypergastrinemia occurred with >6-fold increase in gastrin, similar to that observed in previous series (Table 2) [131,135,137,139,140,141,142,143,144,145,146,147,148,149,150]. The tumoral features (Table 2), which were defined by findings on detailed imaging modalities, as well as surgical exploration in some patients, were also characteristic of the most recent ZES series [103,151,152,153], with most patients having localized disease at presentation, with either primary or secondary lymph node metastases. In addition, the primary tumor was located in the duodenum in the majority of patients and was less common in the pancreas (Table 2), as was originally proposed [123,154,155].

All the ZES patients enrolled in the present study had been treated prior to this study using long-term (mean of 10.2 yrs.) chronic gastric acid antisecretory drugs, and at the time of this study, they were all still being treated with chronic gastric acid antisecretory drugs (Table 3 and Table 4). This result is consistent with other reports on the long-term treatment of ZES patients [79,100,106,109,110,185,186,187,188,189,190,191,192,193,194,195,196,197,198,199,200,201,202,203,204,205,206,207,208,209] due to the following reasons. The majority (80%) of ZES patients cannot be surgically cured, with 20–30% having MEN1/ZES and multiple microscopic duodenal gastrinomas, frequently with lymph node metastases [151,178,210,211]. Thus, the MEN1/ZES patients cannot be cured without a Whipple resection, which is not recommended in most guidelines [103,212]. Furthermore, 30–35% of patients have distant metastases at presentation, which are unresectable, and thus, over all, only 30–40% of the patients with sporadic ZES (75% of all the patients) can be surgically cured [103]. In addition, even in those cured surgically, a high proportion continues to have marked gastric hypersecretion, requiring chronic long-term antisecretory drug treatment [169], which necessitates the constant monitoring of gastric output to appropriately establish the required drug dose, which can change with time [106,213,214]. This makes ZES an excellent model to study the long-term possible side effects of chronic drug-induced gastric acid inhibition, including its possible effect on nutrient absorption such as with VB_12_ (which requires gastric acid secretion for the absorption of food-bound VB_12_), which is an essential vitamin that needs to be absorbed from food [18,52,215]. Prior to this study, 75% of patients had undergone long-term H_2_R treatment at some point (mean of 5.8 yrs.), and 96% received long-term PPI treatment more recently (mean of 5.6 yrs.) (Table 3 and Table 4) because the antisecretory treatment had been frequently started at the onset of symptoms (mean of 3.6 yrs. prior to the study), even before the diagnose of ZES was established (Table 3). The primary H_2_R that the patients had been previously treated with was ranitidine (62%), with 25% treated with cimetidine at some point, whereas of the 96% receiving PPIs (26% without previous H_2_R treatment), the most frequent PPI used was omeprazole (96%), with 15% receiving lansoprazole (Table 4). During the study period (1997–2001), 166 (95%) of patients were treated with PPIs (100% omeprazole), and 5% were treated with H_2_Rs (Table 4). Both with the initial H_2_R dosing (*n* = 9), as well as the H_2_R dose at the time of this study, high daily doses of H_2_Rs were required to control acid secretion (981 ± 86 mg and 1083 ± 391 mg of ranitidine-equivalent daily dose), which is similar to the findings of other studies on ZES patients [79,106,197,198]. Similarly, higher doses of PPIs than are characteristically used in the treatment of patients with idiopathic peptic ulcer disease or GERD [203,216,217,218,219,220] were also initially required to control the acid hypersecretion, as was the case in the present study as well (*n* = 166 pts) (71.4 ± 3.0 and 61.7 ± 3.0 mg/day of omeprazole-equivalent dose) (Table 4), which is a similar result to that reported in numerous other studies on ZES patients [203,216,217,218,219,220]. Before the availability of PPIs, gastric acid-reducing surgery was often performed in addition to the use of H_2_Rs alone [103,146,221,222], which required frequent high dosing to control acid hypersecretion. In older studies, gastric resections were reported to also affect VB_12_ absorption [18,223,224]; so, this also has to be noted in the subsequent analyses. In our study, 11 patients (6.3%) had vagotomies ± Billroth resections prior to being referred to the NIH, and another 22 (12.5%) patients had parietal cell vagotomies at the NIH (Table 3).

### 2.2. Patient Serum VB_12_ and MMA Levels, Plasma tHCY Levels, and Identification of VB_12_-Deficient Patients

**Figure 1:**
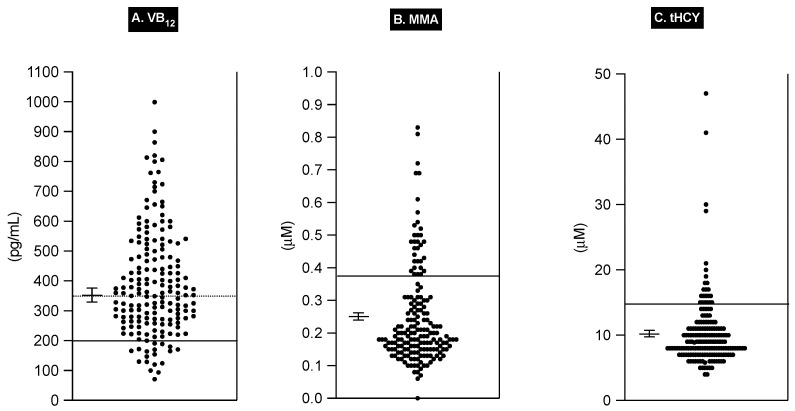
Scatter diagram of serum vitamin B_12_ (VB_12_), serum methyl malonic acid (MMA), and plasma total homocysteine (tHCY) values of 175 ZES patients. Each solid dot represents the value for one of the three parameters for a given patient. The mean ± SEM for all patients is shown for each serum parameter. For VB_12_, the solid line at <200 pg/mL represents a commonly used value proposed for identifying patients with VB_12_ deficiency [112,113,114,115,118,120,233,235]. The VB_12_ dotted line represents the upper limit of the 200–350 pg/mL VB_12_ range, referring to patients with low normal levels of VB_12_, a proportion of whom can be VB_12_-deficient, which can be identified by accompanying blood MMA and/or tHCY levels [115,116,118,232]. In the serum MMA and tHCY panels, the solid line represents the upper limit of the normal level for the assays of 0.37 uM and 0.15 uM for MMA and tHCY, respectively.

The serum VB_12_ levels varied markedly in the 175 patients, with a mean ± SEM value of 394 ± 14 pg/mL and a range of 71 to 999 pg/mL (Figure 1 and Table 5). In various studies, several serum VB_12_ levels have been widely proposed as useful cut-offs for the lower level of normal to identify VB_12_-deficient subjects [118,232]. These include serum VB_12_ levels < 200 pg/mL (148 pmoles/L) [112,113,114,115,116,118,120,233,234] as the most frequently recommended level (sensitivity = 38–39%) [116,232]), as well as other values of 250/270/280 pg/mL [113,115,118], 337/348/350 pg/mL [113,115,116,118], and 1001 pg/mL [115], which have increasing specificity but rapidly decreasing sensitivity [116,118,232].

In addition, serum VB_12_ levels of 200–300 pg/mL [116,233,234,237,239] and 200–350/470 pg/mL [116,215] have been reported to be marginally low levels of serum VB_12_. In our study, 18 patients (10%) had a serum VB_12_ level <200 pg/mL, and another 67 patients (38%) had VB_12_ levels between 200 and 350 pg/mL (Table 5), suggesting that a significant percentage of our patients could be VB_12_-deficient using these criteria.

Unfortunately, in general, it is now generally recognized that no single level of serum VB_12_ alone can identify almost all patients with VB_12_ deficiency [113,114,116,118,120,215,232]. To increase the sensitivity and specificity of the diagnosis of VB_12_ deficiency, two different blood determinations (i.e., the assessment of total homocysteine (tHYC) and methylmalonic acid (MMA) levels) have been developed and are now widely used, either alone or in combination with serum VB_12_ levels, and the combined use of tHYC and MMA is now the recommended approach to diagnose VB_12_ deficiency [84,112,113,114,118,120,215,232]. Both these tests measure the functional effect of VB_12_ (cobalamin) deficiency on metabolic enzymes; the tHCY assay takes into account that methylcobalamin and folate are essential coenzymes for the biosynthesis of methionine from homocysteine, catalyzed by the cytosolic enzyme methionine synthetase, which is essential for de novo nucleic acid biosynthesis, and thus, with a deficiency in either of these coenzymes, there is an accumulation of HCY in the blood [112,113,114,115,116,117,118,120,215,232]. The assessment of blood MMA is even more specific for VB_12_ deficiency because adenosyl cobalamin is essential for the conversion of methyl malonyl-Co-A to succinyl Co-A, catalyzed by methyl malonyl-CoA synthase, which is needed for the proper function of the Krebs cycle and heme biosynthesis, such that VB_12_ deficiency, rather than folate deficiency, results in a buildup of methyl malonyl-CoA, which enters the circulation as free MMA [112,113,114,115,116,117,118,120,215,232]. In the 175 ZES patients, the serum MMA level varied widely, from 0.06 to 0.83 uM (Figure 1 and Table 5). Several upper limits of normal cut-off values for serum MMA have been proposed, including both 0.26 uM [116,118,236,237] and 0.37 uM [115,116,117,118], which are the most commonly used values [115,116,117,118]. In our study, 53 patients (30%) had a serum MMA value > 0.26 uM, and 32 patients (18%) had a serum MMA value > 0.37 (Figure 1 and Table 5). In the 175 patients, the plasma tHCY level also varied widely, from 4.0 to 47 uM (Figure 1 and Table 5). Similar to MMA levels, several upper limits of normal levels for plasma tHCY have also been proposed, with the most frequent being >15 uM [112,114,115,235,240], but a limit >13 uM has also been proposed in several studies [113,115,234]. In our study, 22 patients (13%) had a tHYC level > 13 uM, and 14 patients (8%) had a value > 15 uM (Table 5). In total, 39 patients (22%) had an elevated serum level of MMA > 0.37 or an elevated level of tHYC > 15 uM, with 7 patients (4%) having an elevated level of both. Of the 32 patients having a serum MMA > 15 uM, 7 had a plasma tHYC >15 uM, whereas of the 14 patients with tHCY >15 uM, 7 patients had a serum MMA >15 uM. In numerous studies, it has been reported that both the serum MMA levels and the plasma tHYC levels are very sensitive to alterations in renal function [116,117,238] and that the tHCY levels can also be affected by folate deficiency [113,240]. Neither of these variables was a contributing factor to the serum MMA or plasma tHCY elevations in our patients as all the patients had multiple assessments of serum creatinine as well as serum folate levels, and in all patients, they were within the normal range.

In numerous studies, various combinations of serum VB_12_/MMA and plasma tHYC values have been proposed to better identify patients with VB_12_ deficiencies compared to serum VB_12_/MMA or plasma tHYC alone [112,113,114,115,119,120]. We investigated the results using two of these commonly used criteria in our patients [117,240], which involved identifying patients with a decreased serum VB_12_ level to <200 pg/mL combined with either an elevated serum level of serum MMA (i.e., >0.37 uM) or an elevated level of plasma tHYC (i.e., >15 uM) (Table 5). The result of the combination of VB_12_ and MMA was observed in 14 patients (8%), and the result of the combination of VB_12_ and tHYC was observed in 8 patients (4.6%) (Table 5). Of the 175 patients, one or the other of these two combination criteria was found to be positive in 17 patients (10%), with only the VB_12_/tHYC combination criterion observed in 3 patients, only the criterion of the VB12/MMA combination observed in 9 patients, and both combinations of the VB_12_/MMA and VB_12_/tHCY criteria observed in 5 patients.

**Figure 2:**
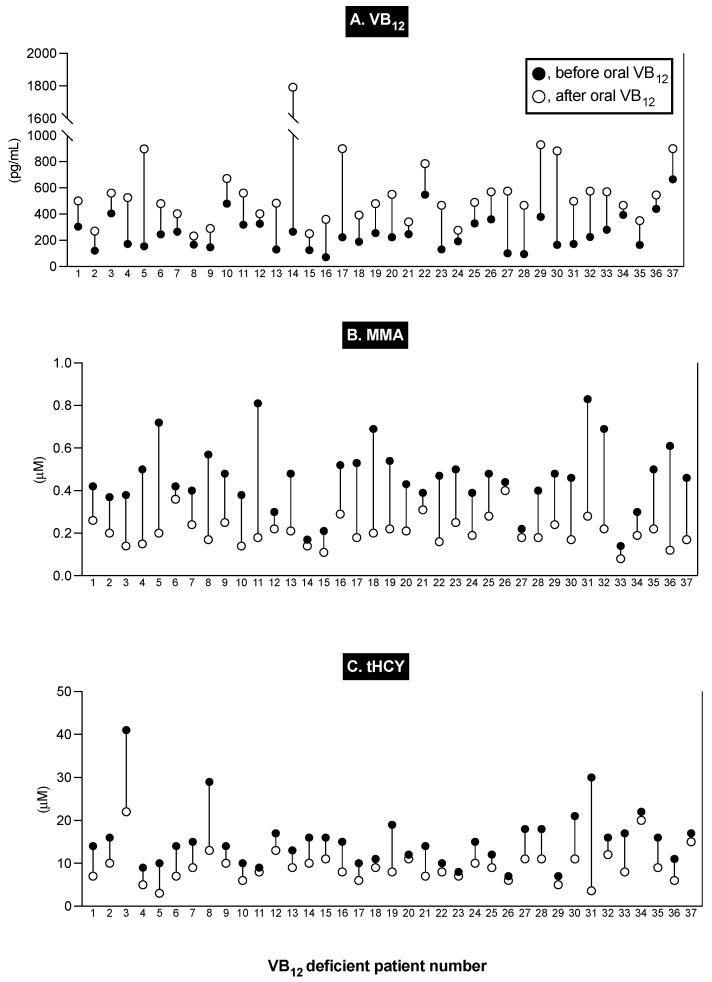
Serum VB_12_, serum MMA, and plasma tHCY results from 37 VB_12_-deficient patients after taking crystalline VB_12_. Patient numbers are shown on the *X* axis, and the change in serum VB_12_, MMA, and tHYC are shown on the *Y* axis. The solid and the open circles represent the serum values before and after taking VB_12_, respectively.

Numerous studies show that to identify all patients with VB_12_ deficiency, the inclusion of a criterion of serum VB_12_ < 200 pg/mL, either alone or in combination with MMA/tHCY levels, has a low sensitivity [113,116,118] because a significant proportion of VB_12_-deficient patients are now known to have serum VB_12_ levels in the range of 200–350 pg/mL or even higher [115,116,118,241]. This is particularly true of patients with subclinical cobalamin deficiency, in which the blood MMA or tHYC identifies VB_12_ deficiency, but the patients are asymptomatic and do not have hematological changes [118,232,240]. One additional criterion frequently used to diagnose VB_12_ deficiency is the assessment of changes in blood MMA/tHYC after the administration of VB_12_, which, in several studies, has been stated to be the single best method of detecting VB_12_ deficiency [112,114,117,236,237,242]. Therefore, we assessed the response to the administration of VB_12_ levels in all the patients with elevated levels of blood MMA or tHCY. Figure 2 shows the results for the 37 patients who met the criteria of having both an elevated blood MMA or tHCY level and who showed an increase in serum VB_12_ level and a decrease in MMA and/or tHCY levels after the administration of VB_12_. For the 37 patients, the mean serum VB_12_ level increased 2.2-fold from 256 ± 22 to 559 ± 47 (*p* < 0.0001), while the mean serum MMA level showed a decrease of 56% from 0.462 ± 0.025 to 0.208 ± 0.011 (*p* < 0.0001), and the mean plasma tHCY showed a decrease of 40% from 15.4 ± 1.11 to 9.30 ± 0.64 (*p* < 0.0001) (Figure 2). This latter combination criterion using two different markers, both widely used to identify VB_12_ deficiency due to an elevated MMA level > 0.37 uM or an elevated tHYC level > 15 uM (with normal renal function and normal folate levels), combined with the appropriate response to the administration of VB_12_ (increased serum VB_12_, decreased serum MMA, or plasma tHCY), was therefore used to identify the 37 ZES patients (21%) with VB_12_ deficiency in our study (Table 5).

**Figure 3:**
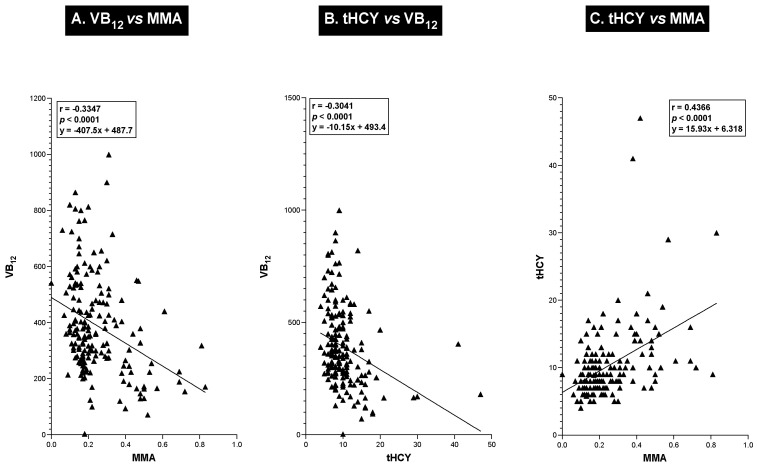
Correlations between patient values for serum MMA and plasma tHYC with serum vitamin B12 levels. Each solid circle or triangle is a value from one patient determined from the same NIH admission. The correlation coefficient, its significance, and the best-fitted regression line equation (using least squares regression analysis) for each correlation are shown.

A correlation analysis of the relationship between serum VB_12_ levels and serum MMA/plasma tHCY levels in a given patient supports the above results that these have a reciprocal relation, with an increase in both blood MMA and tHCY levels as the VB_12_ levels decrease (Figure 3A,B). As seen in Figure 3A,B, there was a highly significant negative correlation (r = −0.304 and r = −0.335) (*p* < 0.0001) between the serum VB_12_ level and the serum MMA level (Figure 3A) or between the serum VB_12_ level and the plasma tHCY level (Figure 3B) in a given patient, and the serum MMA level directly (r = 0.437) and significantly (*p* < 0.0001) correlated with the plasma tHCY level in a given patient (Figure 3C).

### 2.3. Comparison of Vitamin B_12_ Markers (i.e., Blood VB_12_, MMA, and tHCY) and Clinical, Laboratory, and Tumoral Characteristics of ZES Patients with or without VB_12_ Deficiency

Our patients with VB_12_ deficiency had a significantly lower serum VB_12_ level, with more than 40% having a level below 200 pg/mL (*p* < 0.0001) compared to patients without VB_12_ deficiency (Table 6). Furthermore, the VB_12_-deficient patients had a 2.4-fold increase in serum MMA levels (*p* < 0.0001), with 81% exceeding an MMA level > 0.37 uM, all significantly higher than in patients without VB_12_ deficiency (Table 6). Similarly, the plasma tHCY level was increased by almost 2-fold, with almost 40% having a level > 15 uM, which were all markedly increased (*p* < 0.0001) compared to patients without VB_12_ deficiency (Table 6).

The 37 ZES patients with VB_12_ deficiency in our study had no clinical symptoms that indicated the presence of this disorder, and their hematological profile did not differ from the 138 ZES patients without VB_12_ deficiency (Table 7 and Table 8). No patient had an increase in mean corpuscular volume (i.e., MCV > 100 fL), megaloblastic anemia, or reported hypersegmented neutrophils [240]. Therefore, all our VB_12_-deficient patients could be classified as having subclinical cobalamin deficiency, which is commonly observed in older patients, primarily due to food-bound cobalamin malabsorption occurring in up to 40% of patients [113,118,240]. Furthermore, in regard to the clinical/demographic features of ZES, they did not differ between patients with or without VB_12_ deficiency in age, gender, race, presence or absence of ZES symptoms, presence or absence of MEN1, or duration from ZES onset to the time of this study or age at ZES onset (Table 8). Furthermore, the VB_12_-deficient and non-VB_12_-deficient patients did not differ in the magnitude of their original gastric acid hypersecretion (either BAO or MAO), the magnitude of their hypergastrinemia, or the occurrence of previous gastric acid-reducing surgery, including partial gastrectomy (Table 8). In terms of the tumoral features of the gastrinomas, there were no differences between patients with or without VB_12_ deficiency in terms of tumor extent or primary tumor locations (Table 8).

### 2.4. Comparison of Gastric Antisecretory Treatment Characteristics in ZES Patients with or without VB_12_ Deficiency

In our patients with or without VB_12_ deficiency, there were no significant differences in the frequency of previous gastric acid-reducing surgeries, including partial gastrectomies, the frequency of the of use H_2_R or PPI at the time of this study, or the duration of use in the last 10 yrs., although patients with VB_12_ deficiency generally had longer PPI treatment in the last 10 years (*p* = 0.063) (68% vs. 49%) (Table 9, Part I). In patients with or without VB_12_ deficiency, there also was no difference in the ages at which the patients first started gastric acid antisecretory medical treatment or the ages that initial treatment with H_2_Rs or PPIs (Table 9, Part II). While the time of treatment with only an H_2_R prior to the present study was significantly longer in patients without VB_12_ deficiency (*p* = 0.0022), no other antisecretory drug treatment’s duration differed between patients with or without VB_12_ deficiency (Table 9, part III).

### 2.5. Comparison of the Effect of Levels of Control of the Acid Hypersecretion by Gastric Antisecretory Treatment Drugs in ZES Patients with or without VB_12_ Deficiency or on the Biomarkers Used to Determine the Presence of VB_12_ Deficiency (i.e., VB_12_, MMA, and tHCY)

A comparison of the levels of acid control for both the single NIH admission analyzed in detail in the present study (Table 9, Part IV.A) as well as an evaluation of the effect of acid control for all NIH admissions (*n* = 873) over the full five years of this study were performed in our patients with or without VB_12_ deficiency (Table 9, Part IV.B).

For the single admission analyzed in detail, there was a highly significant difference in the two patient groups, with patients with VB_12_ deficiency having a 12-fold lower mean control acid output level (0.14 vs. 1.71 mEq/h) (*p* < 0.0001) (Table 9, Part IV.A), as well as a highly significant difference in the average acid control pH between VB_12_-deficient and non-VB_12_-deficient patients (6.4 vs. 3.7) (*p* < 0.0001) (Table 9, Part IV.A). Furthermore, there was a very large difference in the percentage of patients with a pH value < 3.5 in the VB_12_-deficient/non-VB_12_-deficient groups (0% vs. 56%) (*p* < 0.0001) (Table 9, Part IV.A), which is the pH value that is required to activate pepsin in the stomach [243]; pepsin is essential for the cleavage of food-bound cobalamin to free cobalamin to allow cobalamin (VB_12_) conjugation to R-factor proteins in the stomach, which allows its subsequent absorption [240,245,246,247]. In addition, the reverse pattern for pH > 7 was seen in these acid control values for the single admission analyzed in detail, occurring much more frequently in the patients with VB_12_ deficiency than those without deficiency (49% vs. 8.5%) (*p* < 0.0001) (Table 9, Part IV.A).

**Figure 4:**
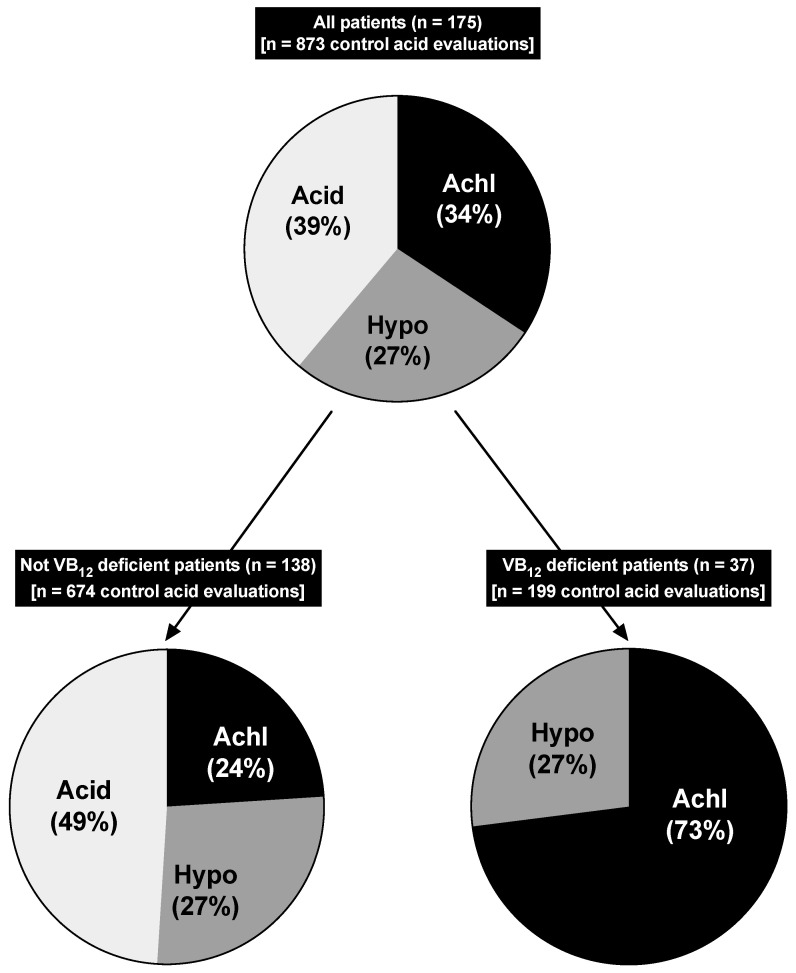
Pie diagram showing mean gastric acid control value acid output category from all patient admissions [*n* = 873] over a 5-year period (1997–2001) stratified by presence or absence of VB_12_ deficiency. As outlined in the Methods section and reported previously [54,244], using the mean acid outputs from gastric acid control analysis from past admissions, all patients were assigned to one of three categories: the presence of sustained achlorhydria (>50% admission acid control = 0) [Achl], sustained hypochlorhydria (acid control levels from 0.1 to <1 mEq/h ->50%) [Hypo], and normal gastric acid in >50% acid controls ≥1 mEq/h [Acid]. The percentages represent the percent of patients in each VB_12_ group [i.e., all pts (*n* = 175); VB_12_-deficient patients (*n* = 37), and non-VB_12_-deficient patients (*n* = 138)], which were in each of the mean acid control categories. For all patients (*n* = 175), VB_12_-deficient (*n* = 37) groups, and non-VB_12_-deficient groups *(n* = 138), there were 60, 27, and 33 patients in the achlorhydric category, 47, 10, and 37 patients in the sustained hypochlorhydria category, and 68, 0, and 68 patients in the acid category, respectively. The presence of achlorhydria was significantly higher in the vitamin B_12_-deficient category of patients (73% vs. 24%) (*p* < 0.001) but not in the hypochlorhydria category (*p* = 0.99), and the presence of normal acid secretion was significantly higher in the non-VB_12_-deficient than VB_12_-deficient patients (49% vs. 0%) (*p* < 0.001).

A similar prominent correlation between the lack of acidity and the development of VB_12_ deficiency was seen in the analysis of the 873 total acid assessments during all admissions for the patients over the full 5-year period (1997–2001) (Table 9, Part IV.B, Figure 4). Similar to previous studies [106,244], this analysis was performed by assigning each patient to one of three acid control categories, which best classify the overall degree of drug-induced acid suppression for each patient for all admissions over this period of time (675 admissions in the non-VB_12_-deficient patients and 199 admissions in the VB_12_-deficient patients). The three overall acid control categories that the patients were assigned to were as follows: sustained achlorhydria, defined as having >50% of all acid control values with an acid output of zero; sustained hypochlorhydria, with >50% of admission with acid output controls of 0.1 to <1 mEq/h; and full acid secretion, with >50% of all admissions having an acid output >1 mEq/h (Table 9, Part IV.B, Figure 4). The presence of sustained achlorhydria was 3 times more frequent in the VB_12_-deficient patients than those without VB_12_ deficiency (73% vs. 24%) (*p* < 0.0001), whereas the opposite trend was seen with the full acid category (>50% admissions >1 mEq/h), which occurred in 49% of all patients with no VB_12_ deficiency but not in any patients with VB_12_ deficiency (*p* < 0.0001) (Table 9, Part IV.B, Figure 4). In contrast to the results in these two acid control categories, there was no difference in the frequency of patients in the sustained hypochlorhydria category with or without VB_12_ deficiency (27% vs. 27%) (Table 9 (Part IV.B) and Figure 4).

**Figure 5:**
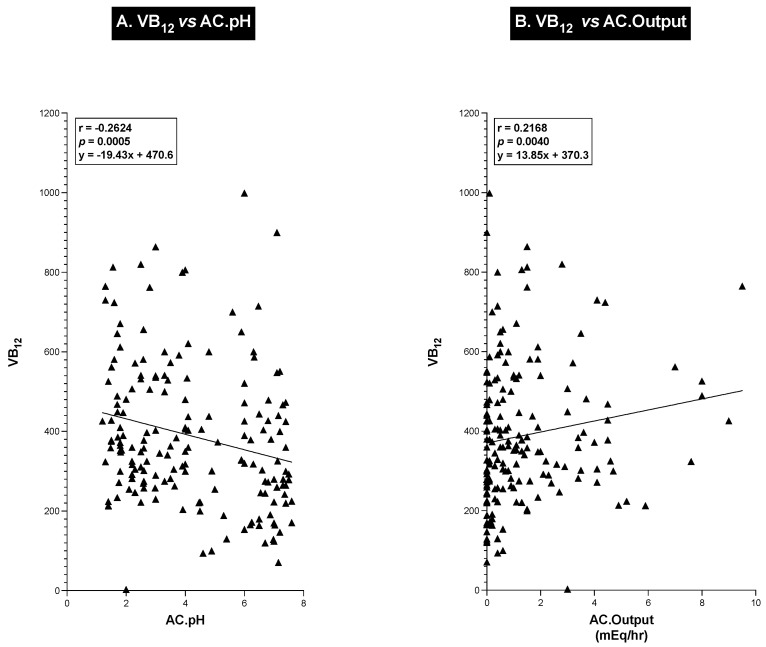
Correlations between patient values for serum vitamin B_12_ levels and gastric acid control values after antisecretory drug intake (gastric acid hourly output after drug intake and pH of hourly gastric control sample). Each solid circle or triangle is a value from one patient determined from the same NIH admission. The correlation coefficient, its significance, and the best-fitted regression line equation (using least squares regression analysis) for each correlation are shown.

**Figure 6:**
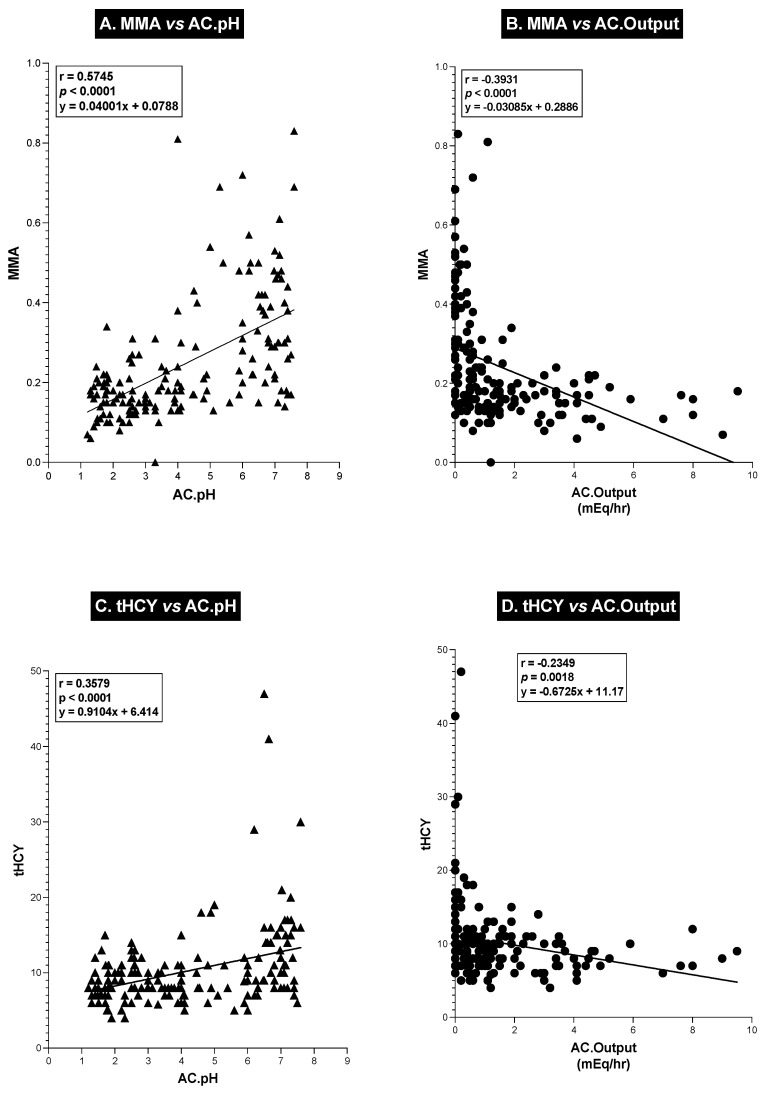
Correlations between patient values for serum MMA and plasma tHYC and gastric acid control (**A**,**C**) values after antisecretory drug intake (pH and acid hourly output after drug intake). Each solid circle or triangle is a value from one patient determined from the same NIH admission. During this admission, the post-drug acid output was determined for the hour prior to the next drug dose, and both the pH (**A**,**C**) and the acid output (**B**,**D**) were in mEq/h were determined and used in the correlations. The correlation coefficient, its significance, and the best-fitted regression line equation for each correlation are shown. The results show highly significant correlations with the increasing serum MMA or plasma tHCY, correlating directly with increased pH (decreasing acidity) of the gastric acid control value (**A**,**C**) and inversely with the acid output value in mEq/h (**B**,**D**).

To further explore the relationship between the antisecretory drug acid control levels [acid output per hour for the hour before the next drug dose and the sample’s acid concentration (pH)] and serum VB_12_ levels, we performed a correlation analysis of these different variables (Figure 5) as well as the relationship between these acid control parameters and serum MMA and tHCY levels in each patient (Figure 6). As shown in Figure 5, the patients’ serum VB_12_ levels showed a highly significant (*p* = 0.0005) negative correlation (r = −0.262) with pH, with decreasing levels of acidity (increasing pH) in the control sample associated with a decrease in serum VB12 levels. Conversely, with acid output, there was a significant direct positive correlation between acid output and serum VB_12_ levels. These results both show that as acid output decreases or with decreasing acidity (pH) of the gastric control fluid, there is a similar proportional decrease in serum VB_12_ levels. A similar analysis with serum MMA (Figure 6A,B) and plasma tHCY (Figure 6C,D) also showed highly significant correlations (*p* < 0.0001) between changes in control acid output (mEq/h) as well as acid concentration (pH). There was a highly significant (*p* < 0.0001) direct correlation (r = 0.574 and r = 0.358) (Figure 6A,C) between both the serum MMA and plasma tHYC levels and the control acid concentration (pH), demonstrating that as the acidity of the sample decreased and the pH of the patients’ acid control samples increased, there was a proportional increase in serum MMA and tHCY levels, which was the opposite pattern seen with changes in serum VB_12_ levels (compare Figure 6,A,C and Figure 5A). Conversely, both serum MMA and plasma tHYC levels (Figure 6B,D) demonstrated a highly significant negative correlation (r = −0.393 and r = −0.235) (*p* < 0.0001, *p* = 0.0018) with changes in control hourly acid output (mEq/h), with increasing hourly acid output (decreasing acid control) associated with decreased blood concentrations of MMA and tHCY, which correlated with the higher serum VB_12_ levels (compare Figure 6B,D and Figure 5B).

### 2.6. Effect of Multivitamin Consumption on Serum VB_12_, Serum MMA, and Plasma tHCY

**Figure 7:**
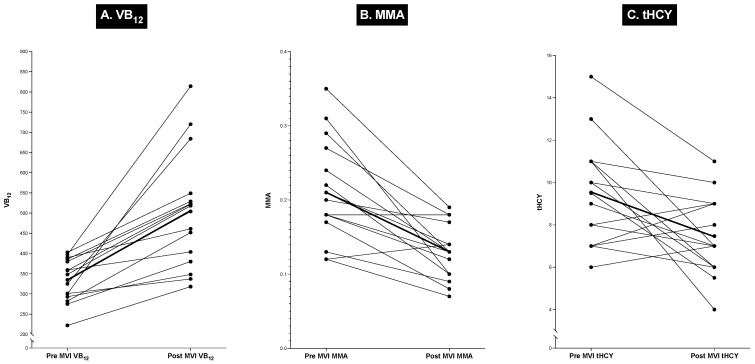
Serum VB_12_, serum MMA, and plasma tHCY results from 15 non-VB_12_-deficient patients after taking multivitamin preparations. The results from 15 patients who had started on multivitamins on their own before their final evaluation are shown. The changes in blood VB_12_, MMA, and tHYC between their last two NIH evaluations are shown on the Y axis, with one being prior to starting any multivitamin and the other after taking a daily multivitamin preparation, all of which contained crystalline VB_12_. Each line shows the effects before or after the multivitamin ingestion. The serum VB_12_, MMA, and tHCY levels for a given patient, shown by the dark lines, represent the mean effect. The mean changes in serum VB_12_ (334 ± 15 to 504 ± 40 pg/mL (*p* = 0.0003)), MMA (0.21 ± 0.02 uM to 0.13 ± 0.01, (*p* = 0.0009)), and tHCY (9.50 ± 0.69 uM to 7.43 ± 0.52 uM (*p* = 0.0176)) are shown.

During this study, the patients did not take multivitamin tablets and were questioned about this in detail during each admission. At the end of our study, 15 patients, none of whom were found to be vitamin B_12_-deficient, started taking standard multivitamin (MVI)/mineral supplements daily on their own. During their subsequent evaluation at the NIH, they were all found to have higher serum vitamin B_12_ levels compared to the previous admission, when they were not taking supplements (Figure 7) [the tables above do not contain information about this or other post-MVI admissions of these patients]. In these 15 patients, after MVI, the mean serum VB_12_ level increased by 34%, from a pre-MVI level of 334 ± 15 to 504 ± 40 pg/mL, which was a significant change (*p* = 0.0003). Similarly, there was a decrease of 38% in the mean serum MMA level, from 0.21 ± 0.02 uM to 0.13 ± 0.01, with 13/15 patients (87%) showing a decrease (Figure 7), which was a highly significant change (*p* = 0.0009). There was more variation in plasma tHCY levels, but for all the patients, there was a 22% decrease, from 9.50 ± 0.69 uM to 7.43 ± 0.52 uM (Figure 7), which also was a significant change (*p* = 0.0176). The results from these patients are included here because they illustrate the importance of studying possible vitamin B_12_ deficiency and the need to carefully evaluate the use of supplemental multivitamin preparations, which, in numerous studies, have been found to be widely used in the US, with an overall frequency of 47–52% [248,249] but up to 80% in some groups, such as patients with cancer [248]. However, their use cannot always be easily established. The importance of this issue will be discussed in more detail in the Discussion section, particularly in relation to the possible contribution to the marked variation in the results of the possible effect of acid suppression drugs on VB_12_ status, as reported in various studies.

## 3. Discussion

This study investigated the effect of long-term gastric acid antisecretory treatment (primarily PPIs) on body VB_12_ status for several reasons. First, there is a continuous increase in the usage of gastric acid antisecretory drugs (especially PPIs) [9,10], both used for established indications such as chronic GERD or peptic ulcer disease and for nonspecific, unapproved indications [9,10,11,14,15], such that they are used by 7–15% of the population, according to various studies, and are among the most prescribed medications [9,10]. In addition, an even larger number of patients are using nonprescribed members of this drug class [12]; so, there is increased concern about the long-term possible risks of this drug class due to its widespread use [9,10,18,19,20,22,23,24,27,28,29,31,32,69]. Second, not only is there increased use of these drugs in the short term but an increasing number of patients are taking them in the long term, especially patients with chronic GERD, such that life-long treatment is becoming increasingly frequent [17]. Third, although these drugs have been reported to be remarkably safe and effective [9,10], there is increased concern about their long-term safety due to their possible risks, especially those observed in numerous epidemiological studies as well as other emerging research [9,10,18,19,22,23,24,25,26,27,28,29,30,31,32,43,44,46,62,63,64,65,67,69,70,73,250,251], in addition to increasing concerns about the effect of chronic hypergastrinemia in humans, which is almost invariably induced by the chronic, long-term use of PPIs [18,36,37,252,253,254]. Fifth, one of these proposed side effects, which has remained contentious, is whether long-term PPI treatment causes various nutrient deficiencies, such as interfering with VB_12_ absorption, and whether its long-term usage can reduce body VB_12_ stores and cause VB_12_ deficiency [18,52,53,54,77,78,79]. This issue is controversial because many studies show that there is likely a long-term treatment effect of PPIs on serum VB_12_ levels and/or causing deficiency [5,54,78,80,81,82,83,84,85,86,87,88,89,90,91,92,93,94,255,256], while several others refute this [79,95,96,97,98,99]. Therefore, the resolution of this issue has important implications for long-term treatment with PPIs in a large group of patients. The fact that the possible effect, if any, of the long-term treatment with potent antisecretory agents such as PPIs on VB_12_ status is still contentious is somewhat surprising because in contrast to several other reported potential side effects of chronic PPI treatment that are receiving considerable attention, the pathogenesis of the possible effect of PPIs on VB_12_ absorption, leading to possible VB_12_ deficiency, if it were to occur, seems clear from prior studies. In contrast, it is unclear which possible mechanism(s) are involved in the PPI-induced increase in the development of dementia, as reported in several studies on chronic PPI use [24,69,257,258,259,260] or whether PPIs result in an increased incidence of bone fractures, particularly of the spine [27,39,44,257,261,262], an increased occurrence of declining renal function and/or increased renal disease [24,27,50,257,263,264], an increased incidence of various infections [250,260,265] or overall mortality [27,65,266,267,268], and their possible pathogenesis [257]. The possible mechanism of PPI-induced VB_12_ deficiency fits well with the results of studies that show that the absorption of VB_12_ from food can be inhibited by any process that markedly inhibits acid secretion [232,247,269,270,271], which is the main basis for PPIs’ clinical effect in acid/peptic disorders [243,247]. This possible side effect is especially a concern with PPIs because they inhibit the gastric parietal cell H+K+ ATPase, one of the most important distal steps in the acid secretory process, and they have a much longer duration of action than histamine H_2_-receptor antagonists such that in most patients, not only are they much more potent at acid inhibition than H_2_Rs but the duration of acid inhibition is markedly longer [197,228,243,272]. VB_12_ is an essential vitamin for humans and needs to be absorbed from animal food sources, in which it is bound to protein [215,240,247,273,274]. To be absorbed, the protein-bound VB_12_ needs to be freed from the protein, which occurs in the stomach by the action of pepsin, a protease [215,247], requiring a pH of <3.5 to be active [243], and then, the free VB_12_ binds to R-protein (haptocorrin) and after the digestion of this complex by pancreatic proteases in the duodenum, the free VB_12_ binds to an intrinsic factor and is absorbed in the terminal ileum [215,269,275,276]. This proposed mechanism is well supported by experimental/clinical results that show that acid-reducing drugs, including PPIs, inhibit VB_12_ absorption in humans [99,215,247,274,277,278,279]. Patients with disease-induced achlorhydria (pernicious anemia and severe atrophic gastritis) develop VB_12_ deficiency [215,240,280], as do patients after total gastrectomy [215,281], total vegans with a strict vegetarian diet [215,282], and patients with inherited disorders disrupting acid production, such as those with inherited disorders of the gastric acid pump due to a defect in gastric H+ K+ ATPase [283].

This study was performed in patients with Zollinger–Ellison syndrome (ZES) for several reasons. These patients have neuroendocrine tumors (gastrinomas) [103], which ectopically secrete gastrin [103,163], resulting in marked basal acid hypersecretion (mean 4-fold increase but can be up to a 15-fold increase) [150] as well as maximal acid secretion [150] because of the trophic effects of gastrin on the gastric mucosa cells (gastric enterochromaffin-like cells and parietal cells) [36,161,189]. Because <25% of all ZES patients are cured by surgical resection (i.e., due to diffuse metastatic disease, multiple tumors as in MEN1/ZES, and recurrent disease after surgery) [100,101,102], the majority of these patients require life-long treatment with gastric acid antisecretory drugs (now, >95% are treated with PPIs) [103,104,105]. In the NIH prospective studies of ZES patients, all the antisecretory drug doses were titrated to reduce the basal acid hypersecretion to <10 mEq/h prior to the next drug dose [104,164]. In ZES patients with advanced GERD [166], previous gastric resections, or with continual symptomatic patients, acid output levels were reduced even further to control symptoms [103,104]. The result of this approach is that all ZES patients treated in this manner at the NIH or other specialty centers [103,105,106] can be used to generate a unique database such that the level of acid secretion on PPIs or other antisecretory drugs is known over a long period of time [103,105,106]. Such an acid secretory database is not available for other diseases, but in ZES patients it’s availability means that acid secretory suppression can be correlated with the possible side effects of PPIs, which might be a result of their profound inhibitory effect on acid secretion, such as its effect on the absorption of nutrients, such as VB_12_. In fact, three previous studies [54,79,244] have used this unique feature of PPI treatment in ZES patients to create an acid secretory database to investigate the effect of PPI acid suppression on patient nutrient body stores (two studies for VB_12_ [54,79] and one for iron absorption [244]). In one study [54], 131 consecutive ZES patients were treated with omeprazole for a mean of 4.5 yrs. (range of 0.2–12 yrs.) and with H_2_Rs for an additional 5 yrs. (range of 0.2–18 yrs.); decreasing serum VB_12_ levels were found with increasing duration of PPI treatment, the magnitude of the decrease correlated with the degree of acid suppression, and eight patients (6%) were found to have a serum VB_12_ level below 200 pg/mL, a level that is common used to suggest the presence of VB_12_ deficiency [112,113,114,115,118,120,233,235]. However, the exact percentage of patients with VB_12_ deficiency was not confirmed because neither blood MMA nor tHYC levels were assessed. In addition, no patient showed clinical symptoms/signs consistent with VB_12_ deficiency, and macrocytosis was seen in only one patient. This study concluded that long-term PPI treatment caused a decrease in serum VB_12_ levels, which, in some of the patients, was similar to levels seen in VB_12_-deficient patients, and that it was directly due to the effect of PPI-induced hypo-/achlorhydria. In the second study [79], 46 ZES patients were studied during treatment with the PPI, i.e., lansoprazole or previously with omeprazole, for a total PPI duration of 11.6 yrs. After 8 yrs., the serum VB_12_ levels started to decrease, without symptoms of VB_12_ deficiency or hematological changes, and by assessing the response of the serum MMA/tHCY level to the administration of VB_12_, 31% of the patients were reported to have developed VB_12_ deficiency. However, in contrast to the first study [54], it was concluded that these changes in serum VB_12_ levels/stores were not due to PPI-induced changes in acid secretion because the changes in acid secretion were not prolonged or profound enough to explain those seen serum/body VB_12_ changes. Therefore, these two previous studies in ZES patients [54,79] did not explain the effect of chronic PPI treatment on serum/body VB_12_ levels/stores or the possible role of PPIs’ effect on acid secretion for any observed changes and gave mixed results, similar to those obtained in the large number of studies reviewed above in non-ZES patients, which attempted to determine the effect of PPIs on VB_12_ levels/stores and the role of PPI-induced acid suppression regarding any observed changes, resulting in equally divided conclusions.

The present study was designed to attempt to resolve the issue of whether long-term, chronic PPI use could affect serum VB_12_ levels in ZES patients, which could lead to VB_12_ deficiency, and whether this was mediated by the effect of PPIs on gastric acid suppression. To carry out this study, we included a larger number of ZES patients (*n* = 175) than the previous studies reviewed above [54,79] because of our experience with previous ZES studies [103,169,184,198]. Previous studies have demonstrated that there could be a wide variation in PPI-induced acid suppression not only between patients but also in a given patient over time, which could be influenced by adjustments in PPI dosage/and or frequency, as well as aspects of the ZES itself (tumor growth, level of gastrin ectopic release, hormonal aspects such as serum calcium levels in MEN1/ZES patients, tumor load changes after surgical resection, treatments against advanced disease, etc.) [103,169,184,198]. We extended this study over a 5-year period (1997–2001), with yearly admissions and assessments of tumor activity by detailed imaging studies [102]. Furthermore, during each assessment, we conducted a careful review of whether any oral VB_12_ supplements were being used and determined serum VB_12_, serum MMA, and plasma tHCY levels at all admissions with appropriate controls (assessment of renal function and serum folate levels). In addition, other studies were regularly performed, including hematologic studies and an assessment of gastric acid secretory control at that admission. Lastly, to clearly identify patients with proven VB_12_ deficiency, we measured the blood level of either MMA or tHCY, which are established markers of body VB_12_ deficiency [112,113,114,115,116,117,118,119,120], as well as a response toward an elevated MMA/tHCY level after the administration of oral VB_12_ supplements, which is considered by many to be the most sensitive measurement of VB_12_ deficiency [112,114,117,236,237]. The increased number of patients and patient admissions/assessments over time allowed us to collect sufficient data to systematically assess all clinical, laboratory, and tumoral factors, PPI treatment, and acid secretory controls that could affect VB_12_ blood and/or VB_12_ body stores in the patients and to perform appropriate correlative analyses to gain insights into any effects found on VB_12_ levels/stores in our patients.

Our results strongly support the conclusion that in our patients, the chronic, long-term use of antisecretory drugs, particularly PPIs, has a prominent effect on the serum VB_12_ levels as well the VB_12_ body stores, with the development of VB_12_ deficiency in 20% of the patients. First, in our study, the mean serum VB_12_ level was 394 ± 14 pg/mL, with a mean value of 390 ± 14 in those taking PPIs, which is significantly lower (*p* = 0.03) than that in the patients taking only H_2_Rs in the present study (502 ± 44); it was also significantly lower (*p* < 0.001) than the value reported in 20 ZES patients undergoing long-term treatment with only ranitidine in our previous study using the same VB_12_ assay (582 ± 63 pg/mL) [54]. These results are consistent with two previous studies on ZES patients [54,79], which reported that chronic antisecretory treatment with PPIs decreased serum VB_12_, with a decrease of 53% in one of the studies [54] over a 10-year period. Second,18% of our patients had a serum VB_12_ < 200 pg/mL, which is commonly used in VB_12_ studies to indicate that a given patient likely has VB_12_ deficiency [112,113,114,115,118,120,233,235], and 38% of our patients had a serum VB_12_ level of 200–350 pg/mL, which was considered a low level of serum VB_12_ in numerous studies [115,116,118]. Third, 18% of our patients had an increased serum MMA level of >0.37 uM, which is a frequently used criterion to identify the presence of VB_12_ deficiency [115,116,117,118] because its elevation shows a metabolic insufficiency of VB_12_ body stores to carry out a critical VB_12_-dependent metabolic pathway [115,116,117,118,235,236,237]. Similarly, 8% of all patients had an elevated total homocysteine (tHCY) plasma level (i.e., >15 uM), which, in the presence of normal renal function and a normal serum folate level, is commonly used as another criterion for the diagnosis of VB_12_ deficiency [112,114,115,235] because its elevation also shows a metabolic insufficiency of VB_12_ body stores to carry out a critical VB_12_-dependent metabolic pathway [112,113,114,115,234]. Fourth, with the administration of oral crystalline VB_12_ supplements, each of the 37 patients (21%) with either an elevated blood MMA or tHCY level showed a concomitant increase in serum VB_12_ levels combined with a decrease toward normal levels of both the blood MMA and tHCY levels, which are considered by many to represent the most sensitive measurements of VB_12_ deficiency [112,114,117,236,237]. Fifth, we observed that the decreasing serum VB_12_ levels are in fact closely coupled with the increases in blood MMA and tHCY levels, shown by a highly significant (*p* < 0.0001) inversed correlation of serum VB_12_ levels with both blood MMA and tHCY levels. Sixth, the claim that the development of VB_12_ deficiency is due to the prolonged chronic use of PPIs is supported by our finding that none of the patients undergoing long-term treatment with only H_2_Rs developed VB_12_ deficiency, as shown by significantly higher serum VB_12_ levels in the latter group of patients and by our previous study [54], which showed significantly higher serum VB_12_ levels in ZES patients chronically treated with H_2_R than those treated with PPIs. Furthermore, in our previous study [54], a significantly higher proportion (*p* < 0.001) of patients who had received long-term treatment with PPIs compared to those receiving long-term treatment with H_2_Rs had serum VB_12_ levels < 200 pg/mL, which is a criterion widely used to identify a patient with VB_12_ deficiency [112,113,114,115,118,120,233,235]. These results strongly support the conclusion that ZES patients who received long-term treatment with PPIs developed a decreased serum VB_12_ level, which increased with the duration of treatment [54,79], potentially resulting in VB_12_ deficiency. Furthermore, it directly establishes the mechanism by which the PPI causes decreased body stores of VB_12_ in these patients.

One could propose several different mechanisms through which PPIs could interfere with VB_12_ absorption, including that PPIs inhibit acid secretion to such an extent that it interferes with its essential role for VB_12_ absorption from the typical diet, which has been extensively shown in studies on other acid-reducing disorders [54,85,247]; that PPIs interfere with the secretion of the intrinsic factor (IF), which per se is needed for the efficient absorption of VB_12_ after VB_12_ is released from R-factors by pancreatic protease digestion and then complexes with IF, which is essential for the specific receptor-mediated absorption of VB_12_ in the ileum [247,284]; that bacterial overgrowth develops or results in VB_12_ deficiency [247,285,286], which could occur due to bowel dysfunction due to the frequent GI surgeries these patients had or due to the presence of hypo-/achlorhydria [247,285,286]; due to previous gastric resections or other gastric acid-reducing surgeries that many of these patients frequently had prior to the availability of potent medical acid secretory drugs [113,224,247]; due to a direct effect of PPI-induced hypochlorhydria on the absorption of VB_12_; due to the development of atrophic gastritis or pernicious anemia, each of which is frequently associated with VB_12_ deficiency, perhaps due to *H. pylori* infections, which are common found in elderly patients with severe VB_12_ deficiency [233,247,287]; due to a specific ZES tumor variable such as an effect of hypergastrinemia and tumor-induced cachexia; due to anti-tumor treatments in patients with advanced disease; or due to the tumor’s location, which induces intestinal dysfunction, with effects on VB_12_ absorption. Many of these possibilities can be excluded based on the known actions of PPIs, whereas many of the others can be excluded using the detailed analyses of these variables, which were performed in this study or other studies. Previous studies demonstrated that PPIs do not alter IF secretion [288,289], that gastrinomas do not alter IF secretion by inducing hypergastrinemia, and that in fact, ZES patients are hypersecretors of IF [275]. No cases of blind loop syndrome with bacterial overgrowth in ZES patients have been reported in the literature even though these patients received abdominal surgeries very frequently, especially in the past, and furthermore, in our series of more than 400 ZES patients, we did not have any cases with this diagnosis. Also, there is no evidence in the literature that hypergastrinemia per se affects vitamin B_12_ absorption or that hypochlorhydria per se affects free VB_12_ ‘s absorption. Although hypo-/achlorhydria has been shown to have a marked effect on the absorption of food-bound VB_12_, it had no effect on the absorption of crystalline VB_12_ in several ZES patients [54,290,291,292]. However, in one study [293], when the duodenal pH was very acidic in a ZES patient, VB_12_ absorption was impaired; however, when the pH was raised to neutral (pH 7), in the same patient, the absorption of VB_12_ was normalized. While there are occasional case reports of the development of atrophic gastritis in ZES patients [290,294,295,296], in some cases, after acute gastrointestinal infections [296,297,298], this is a rare occurrence, and in our series of 400 patients, we did not see any cases of this. Many of the proposed possible explanations for the PPI-associated VB_12_ malabsorption that are specifically related to features of ZES can be excluded by the analyses performed in the present study, especially when combined with the results of other studies. In particular, no clinical feature (age, gender, duration of disease, presence of ZES symptoms, or presence of MEN1) or laboratory feature (level of BAO, MAO, or fasting gastrin) was correlated with the presence or absence of VB_12_ deficiency (Table 8). A particularly important finding is that the presence or absence of a history of any surgical gastric acid surgical procedure [223] had no effect on the development of VB_12_ deficiency in our study (Table 8), even though in numerous studies that included a follow-up of non-ZES patients who underwent such surgical procedures, especially after gastric resection, there was an increased occurrence of VB_12_ deficiency [113,224,247]. Similarly, there was no specific tumoral feature, including the location of the primary gastrinoma, or extent of the disease, including patients with extensive liver metastases, many of whom underwent anti-tumor therapies. These results agree with our previous study [54], which showed a correlation between the serum VB_12_ levels in ZES patients and chronic long-term treatment with PPIs and/or H_2_R gastric antisecretory therapy, with similar clinical, laboratory, and tumoral variables to those assessed in the present study, both showing that the presence or absence these ZES variables in any of these areas did not correlate with the effect of antisecretory therapy on serum VB_12_ levels either positively or negatively. The above analysis provides evidence to exclude all of the above possibilities, except the first mechanism proposed above, which is that PPIs inhibit acid secretion to such an extent that it interferes with its essential role for VB_12_ absorption from the typical diet, which has been extensively shown in the literature for other acid-reducing disorders [54,85,247].

Many results of the analyses of the relationships between their drug gastric acid secretory control rates and their various body VB_12_ measurements, including their VB_12_ serum levels, VB_12_ body stores, and the presence or absence of VB_12_ deficiency in the individual patients, provide direct evidence in support of the proposed conclusion that PPI-induced sustained hypo-/achlorhydria directly results in decreased VB_12_ body stores in our patients, which led to the development of VB_12_ deficiency in some of our patients, according to each of the criteria used (MMA, tHCY, and response to VB_12_ supplementation). First, at the time of the admission, a highly significant (*p* < 0.0001) direct correlation was found between the individual patients’ serum VB_12_ level and the control acid output on the drug for the last hour prior to the next drug dose, whereas with the acid control pH, the opposite trend was seen, with a highly significant (*p* < 0.0001) inverse relationship between the acid fluid pH and the serum VB_12_ levels. This relationship strongly supports the conclusion that in these patients, as the gastric acid output decreased due to a greater inhibitory effect of the PPI, coupled with the decreased acidity, resulting in an increased gastric pH, there was a decrease in the level of the serum VB_12_ over time. We conclude that this was due to the PPI based on the finding that the serum VB_12_ levels were significantly higher in the patients being treated with only H_2_Rs and that none of the patients treated with only H_2_R developed VB_12_ deficiency, whereas the lower levels of serum VB_12_ in the chronic PPI-treated patients resulted in 22% of these patients developing VB_12_ deficiency. This conclusion is also supported by our previous study [54], which demonstrated that drug acid control rates were 3-fold higher in patients with chronic long-term treatment with only an H_2_R compared to those treated with PPIs; the percentage with stringent acid control (<1 mEq/h) was significantly less, and the VB_12_ levels were significantly higher. Second, not only were the changes in serum VB_12_ levels closely correlated with the changes in the gastric acid control levels but there was also a highly significant (*p* < 0.0001) and close correlation between the levels of blood MMA and tHYC levels and both the control acid output levels as well as the acidity (i.e., pH) of the sample, with the correlations showing an opposite trend compared to the changes in the serum VB_12_ levels. This occurs because as the gastric output is reduced, coupled with reduced acidity (increasing pH), lower VB_12_ levels and, over time, VB_12_ deficiency develop, resulting in increasing MMA/tHCY levels, which change in the opposite direction compared to VB_12_ levels because they directly monitor the adequacy of body VB_12_ given that the enzyme’s activity responsible for the cascade of metabolism involving these substances directly depends on the availability of VB_12._ Thus, as VB_12_ stores decrease, both blood MMA and tHCY levels increase [115,116,117,120,232,233,240]. Third, the mean drug control acid output for the hour prior to the next dose of antisecretory drug during the admission was 12-fold lower in patients with VB_12_ deficiency compared to those without VB_12_ deficiency, with the result that the gastric pH was 2-fold higher and none of the VB_12_-deficient patients had a gastric acid control pH < 3.5, which is the level required for the activation of gastric pepsin [243]. This result supports the conclusion that the mechanism of gastric acid hyposecretion/achlorhydria causes a decrease in serum VB_12_ levels, which resulted in the development of VB_12_ deficiency in our patients. VB_12_ deficiency occurred because free VB_12_ could not be released from the food-bound form in the stomach by the action of gastric proteases, which require this degree of acidic pH, and as a result, free VB_12_ could not be generated to allow its subsequent binding to gastric R-factor proteins [215,245,246,247] and, later, the intrinsic factor, which are all required for its efficient absorption [215,247,269,275]. Fourth, the conclusion for the central role of the profound inhibition of gastric acid secretion mediating the VB_12_ changes in our patients is also supported by the analysis of all the gastric acid controls from the patients’ yearly admissions (*n* = 873) over the 5 years of this study. This showed that a 3-fold higher percentage of patients in the VB_12_-deficient group of patients had sustained achlorhydria than the patients without VB_12_-deficient (75% vs. 24%), whereas the reverse was true in the non-VB_12_-deficient patients, with half showing high acid controls, whereas none of the VB_12_-deficient patients had high acid controls. Therefore, the results of our study provide strong support for the conclusion that chronic, long-term treatment with PPIs can result in decreasing serum VB_12_ levels, which, in time, can further result in VB_12_ deficiency, mediated by PPI-induced acid hyposecretion, resulting in chronic food-mediated VB_12_ malabsorption.

In conclusion, one could raise the question of whether the findings in this study on ZES patients that received prolonged treatment with PPIs can have sufficient hyposecretion of gastric acid secretion to cause VB_12_ deficiency and whether it can provide any insights into the much larger and still unanswered and controversial question of this possible long-term effect of chronic PPI treatment in patients with idiopathic GERD. One could argue the answer is clearly no because the patient populations and treatment strategies are very different in several ways. First, although there is a more recent attempt to lower the PPI daily dose in ZES patients [106], our ZES patients’ mean daily dose of PPIs was higher (60 mg/day omeprazole-equivalent dose) than that generally used in most patients with idiopathic GERD [3,299]. Second, 71% of our patients required more than a daily dose of PPIs (i.e., BID or TID), which is more frequent than that of patients in the series with idiopathic GERD, in which this percentage is usually less than 30% [300,301]. This is an important difference because numerous studies have shown that increasing a single daily PPI dose results in less acid suppression and symptom control than splitting the same dose not only in ZES patients [106,228,302] but also in patients with idiopathic GERD [301,303]. Third, many patients with ZES have a prolonged history of the disease before the diagnosis is made (4–7 yrs.), during which time their gastric acid hypersecretion is often inadequately controlled, which can lead not only to persistent UGI acid/peptic symptoms but also to malabsorption, including for VB_12_ [293], which, when combined with a less-than-optimum diet due to general ill health, can lead to lower body VB_12_ body stores, at the time that adequate acid control measurements are started. Therefore, this could lead to a shortened time to develop even lower levels of body VB_12_ if the acid hypersecretion is over-controlled due to PPI-induced achlorhydria. Fourth, a similar logic to that in the third point described above is found in the subsequent natural history of patients with ZES after diagnosis, which separates their probability of developing low VB_12_ body stores and/or VB_12_ deficiency from patients with idiopathic GERD, who are usually healthier. This occurs because, after diagnosis, many of these patients undergo various procedures/treatments (surgeries, treatment of advanced disease, and surgeries/treatments for those with MEN1/ZES) [103] that have extended periods of poor nutrition. However, the present study demonstrates these results in humans for the first time, with correlative acid measurements showing that in at least one group of patients, i.e., those with ZES, PPI-induced acid hyposecretion can result in low body VB_12_ stores and VB_12_ deficiency, using three different widely used criteria of VB_12_ deficiency, and thus resolves the controversy of whether PPI suppression of acid secretion is responsible for the decrease in serum VB_12_ levels in this group of patients, as previously reported in these patients in two studies [54,79]. Whether similar findings to those in our patients might be found in patients chronically taking PPIs for other diseases, such as GERD, can be determined by a similar protocol to ours in the other groups of patients. However, it is unlikely that this will ever be investigated because gastric acid measurements have largely disappeared from all but a few centers; therefore, the type of correlations we performed in this study will be very difficult to complete. On the other hand, approximately half of the studies on patients with chronic, long-term PPI treatment in other diseases (primarily GERD) support the conclusion that a similar process may occur with PPI treatment in these patients [54,78,80,81,82,83,84,85,86,87,88,89,90,91,92,93,94], while the other half of the studies do not support this conclusion [79,95,96,97,98,99]. The explanation for these different results is not currently clear, although our studies provide one insight that may have been overlooked in these studies and that could contribute to the differences in the results. This insight was that at the end of our study, in 15 patients, the blood VB_12_ levels began to rise, with a concomitant decrease in blood MMA and tHCY levels, although none of these patients had reached levels where they were defined as being VB_12_-deficient. In each case, this turned out to be due to the use of multivitamins obtained by the patient themselves without any doctor’s prescription but instead obtained over the counter. Even though, whenever possible, we asked the patients and their spouses detailed questions about the use of multivitamins or vitamin supplements upon every admission, which our patients denied taking throughout this study, we still did not find any group of patients that had started taking multivitamins on their own; the patients stated that they did not take multivitamins when questioned. Multivitamins are now used in 47–52% of the population [248,249] and up to 80% in patients with cancer [248], and as can be seen in our study, their use can sometimes be difficult to identify. Our result shows that in these 15 patients, a moderate amount of crystalline VB_12_ in the standard multivitamin (7–55 ug/tablet) that our patients were taking was enough to reverse the changes in VB_12_, blood MMA, and tHCY levels induced by chronic PPI use. This result is comparable to the reports of the effect of standard multivitamin use in various patient groups, including patients with diabetes, which resulted in an increase in serum VB_12_ levels and a correction of the presence of VB_12_ deficiency manifested by reversing the elevated blood levels of MMA and tHCY [241,304,305,306,307]. It is not clear whether this contributes to the differences in results regarding PPI-induced effects in other diseases studied, which reported differing effects of chronic PPI use on VB_12_ levels/body stores. However, as seen in our study, any intermittent or even regular use of multivitamin preparations can be difficult to detect or exclude. This possibility is supported by studies that show sensitivity for the detection of the use of dietary supplements by self-reporting, which was found to be 66%, 69%, and 75% in three studies, thus suggesting that it can frequently be underestimated [276,308,309]. Furthermore, at least one study [87] examining the effect of PPIs on serum VB_12_ levels in elderly patients concluded that the failure to detect a PPI effect might be because 41% of the patients were taking multivitamin supplements. Considering that in the US, patients can easily buy MVIs without prescriptions, it is very difficult to completely exclude periodic MVI use in any study.

## 4. Materials and Methods

The patients in this study are part of an ongoing NIH prospective study on various aspects of Zollinger–Ellison syndrome (ZES) that started in 1974, as approved by the clinical research committee of the National Institute of Diabetes, Digestive and Kidney Diseases of the National Institutes of Health (NIH), the characteristics of which have been described previously [100,150,163,310]. All subjects gave their informed consent for inclusion before they participated in this study. This study was conducted in accordance with the Declaration of Helsinki, and the protocol was approved by the clinical research committee of the National Institute of Diabetes, Digestive and Kidney Diseases of the National Institutes of Health (NIH). This cohort includes all patients with established ZES who had acid antisecretory treatment with either H_2_Rs or PPIs and who had their antisecretory doses set according to the results of gastric acid testing as described previously and reviewed briefly below [166,227,228].

All patients who were diagnosed with Zollinger–Ellison syndrome (ZES) without a total gastrectomy at the National Institutes of Health from 1997 to 2001 or who were taking gastric antisecretory drugs because they were not surgically cured were eligible [106,168,225]. The diagnostic criteria for ZES were as previously described, which involved demonstrating inappropriate hypersecretion of gastrin by demonstrating hypergastrinemia in the presence of acid hypersecretion, positive provocative testing with either secretin or calcium, a positive histologic diagnosis of gastrinoma, or a combination of these as described previously [103,150,310,311]. To accomplish the diagnosis, all patients underwent nasogastric aspiration, a basal acid output (BAO), and pentagastrin-stimulated maximal acid output (MAO) when initially assessed, which were performed as described previously [104,150]. Also, they all underwent gastrin provocative testing (all had secretin testing and, if equivocal, calcium provocative testing) as previously described [163,310] as well as multiple fasting gastrin tests, some of which were performed without the administration of any antisecretory drugs, as described previously [104,163]. Serum gastrin analysis was performed by Bioscience Laboratories (New York, NY, USA), and all samples were diluted into the normal range for accurate determination [213]. The possible presence of MEN1 was investigated in all patients using the assessment of family history, in addition to serum/plasma parathormone, prolactin, and ionized calcium assays as described previously [100,101,156,161].

All patients were initially assessed for tumor location and tumor extent using multiple tumor localization approaches, including imaging studies performed as previously described, including computed tomography scan with contrast (CT scan) [172,312], magnetic resonance imaging with contrast (MRI) [313], somatostatin receptor imaging [314,315], and transabdominal ultrasonography. In patients with equivocal results, a proportion also underwent selective celiac angiography with contrast, with or without secretin-stimulated gastrin sampling or portal venous gastrin sampling [177,316,317].

All patients were assessed for possible surgical cure, except patients with MEN1/ZES with imaged tumors < 1.5–2 cm [102,167] or those with advanced unresectable disease, who were instead assessed for the treatment of metastatic disease (chemotherapy, interferon, or somatostatin) as described previously [103,176,184].

### Study Design

The primary purpose of this study was to assess ZES patients who require life-long gastric acid antisecretory agents for possible vitamin B_12_ deficiency by assessing serum methylmalonic acid (MMA), total homocysteine (tHCY), and VB_12_ levels and to provide insights into its possible causative factors, especially the role of the long-term suppression of gastric acid hypersecretion. Possible causality was assessed by correlating the patient’s VB_12_ status to various possible contributing clinical, laboratory, tumoral, and treatment features of the ZES patients. After having their gastric acid hypersecretion controlled for at least 6 months, consecutive patients with ZES were enrolled in this study. Prior to this study, all patients entering this study, who had their gastric acid hypersecretion initially controlled long-term before 1983, were treated with a histamine H_2_-receptor antagonist (H_2_R) (cimetidine or ranitidine), either alone or with an anticholinergic agent [106,198,214,225,226]. After 1983, in almost all cases, patients were initially treated with a more potent H_2_R antagonist (famotidine) [214,227], switched to a PPI, or initially started on a PPI (omeprazole or lansoprazole) [197,214,228].

The maintenance dose of antisecretory drugs was established in all cases by drug dose titration based on gastric acid antisecretory measurements for the hour prior to the next drug dose, determined as described previously [104,106,164,214]. The primary criterion for the effective control of acid hypersecretion was to reduce the acid output to <10 mEq/h prior to the next dose [104,164,214], which has been shown to allow the healing of mucosal lesions, as well as prevent additional acid-induced lesions in ZES patients [104,164,214]. However, in patients with moderate to severe GERD [166] or a previous partial gastrectomy, acid reduction to lower levels (<5 mEq/h or less) is frequently required to completely control all symptoms and result in complete mucosal healing. A similar approach was used with both H_2_Rs and PPIs with stepwise dose adjustment after starting with 150/600 mg every 6–8 h for cimetidine, 150/300 mg with ranitidine, 20/40 mg of famotidine, or a once-daily dose of omeprazole/lansoprazole of 60 mg [104,106,164,214]. The initial daily dose of PPIs, which were the most frequent antisecretory drug used during this study period (1997–2001), was increased by 20 mg/day (15 mg for lansoprazole) until acid output was controlled; if not, the daily dose was increased to 120 mg/day, and the dosage was split to 60 BID and increased further as needed [106,214]. A prior study established that the relative potencies for famotidine/ranitidine/cimetidine (i.e., H_2_Rs) in ZES patients are 1:9:32 [227], and for PPIs, these values are as follows: omeprazole (20 mg) = 40 mg esomeprazole, 30 mg lansoprazole, 40 mg pantoprazole, and 20 mg of rabeprazole [231]. Using these equivalent doses, a ranitidine-equivalent dose or omeprazole-equivalent dose was calculated for each patient to allow the comparison of drug dosing in different patients on different drugs, as described previously [106].

Upon entering the present study and during each subsequent yearly evaluation at the NIH (1997–2001), a complete medical history and a complete physical examination were performed to assess for any symptoms/signs that might indicate VB_12_ deficiency. In addition, a complete blood count, a complete clinical chemistry profile (creatinine, BUN, liver function studies, and serum electrolytes), and the levels of fasting serum gastrin, serum folate, serum vitamin B_12_, serum MMA, and serum tHCY were determined by the NIH Clinical Center Hematological and Clinical Chemistry laboratories. During all visits, patients were questioned about the use of any multivitamin preparations to ensure that none were being taken. Serum vitamin B_12_ and serum folate levels were measured by radio-immunoassays (Ciba-Corning Co., Medford, MA, USA). The inter-assay variation for the serum vitamin B_12_ and serum folate levels for four different standards was as follows: 140 pg/mL (8%), 387 pg/mL (6%), 681 pg/mL (8%), and 964 pg/mL (10%) for vitamin B_12_ and 1.5 ug/mL (11%), 3 ug/mL (9.8%), 5 ug/mL (6.4%), and 11 ug/mL (7.5%) for serum folate levels. In the patients who were found to have low vitamin B_12_ serum levels, anti-parietal and anti-intrinsic factor antibodies were obtained, which were negative in all patients tested. All hematology measurements were obtained with the National Institutes of Health Clinical Chemistry/Hematology Laboratories. Serum MMA levels were measured through the National Institutes of Health Clinical Chemistry laboratory by gas chromatography/mass spectrometry, with the normal level being <0.37 uM, and plasma tHCY levels were measured using the Abbott IMx immunoassay and assessed using a normal level < 15 uM. Patients were classified as VB_12_-deficient if they had an elevated serum MMA level or tHYC level in the presence of normal serum folate levels (>3.0 ug/L) and a normal serum creatinine level; and the serum MMA and tHYC levels decreased toward normal levels after taking oral crystalline VB_12_ (50 ug BID). Patients were classified as having subclinical cobalamin deficiency (SCCD), as defined previously [113,232,234], if they had compromised cobalamin metabolism, as defined by abnormal MMA and/or tHCY levels without folate deficiency/altered renal function, and were asymptomatic and nonanemic. Patients diagnosed with SCCD were treated with crystalline V_12_ supplements (primarily oral 50 ug BID crystalline cobalamin) and reassessed to confirm the normalization of MMA and tHCY levels with elevated VB_12_ levels.

Gastric acid secretory drug control rates were reviewed for all admissions over the 5-year study period. Based on their acid secretory rate over all the evaluations during the study period, patients were classified into different acid control categories, similar to other studies on ZES patients [54,106,244]. Three different acid control categories were identified: sustained achlorhydria (>50% admission acid control = 0), sustained hypochlorhydria (acid control levels from 0.1 to <1 mEq/h (>50%), and normal secretion with >50% acid controls ≥1 mEq/h.

The results of the single admission over the 5-year study period, which showed the most advanced MMA/tHCY changes, were used to classify the patient as VB_12_-deficient or non-VB_12_-deficient, and MMA/tHCY and VB12 levels as well as the acid secretory data during this admission were used for correlative and comparative analyses.

The patient variables used in comparative analyses to identify possible contributing factors to the development of VB_12_ deficiency, with correlations with VB_12_, MMA, and tHCY levels, including those for clinical characteristics and disease course, tumoral characteristics, and laboratory characteristics, including acid secretion, duration dosage, and the type of previous acid secretory treatments, are defined in the tables above.

Statistical analysis was performed using the Mann–Whitney–Wilcoxon test and the Fisher’s exact test. Least squared regression analysis was used to calculate correlation coefficients, with *p*-values < 0.05 considered significant. All the continuous variables are reported as mean ± SEM.

## Figures and Tables

**Table 1:** Patient clinical characteristics and disease course.

Characteristic	Number (%)
Patient number	175 (100%)
MEN 1 present (a)	45 (26%)
Gender	
Male	91 (52%)
Female	84 (48%)
Race	
White	137 (78%)
Black	29 (17%)
Hispanic	6 (4%)
Asian	3 (1%)
Age at ZES onset (yrs.) (b)	
Mean ± SEM	39.9 ± 0.9
(Range)	(12.9–65.0)
Age at ZES diagnosis (yrs.) (c)	
Mean ± SEM	45.8 ± 0.9
(Range)	(14.2–70.6)
Age at 1st NIH visit	
Mean ± SEM	47.8 ± 0.9
(Range)	(13.1–71.0)
Age at the time of present study	
Mean ± SEM	53.8 ± 0.9
(Range)	(21.3–81.8)
Presenting clinical symptoms/features (d)	
Pain	135 (77%)
Ulcer history	121 (63%)
Diarrhea	140 (80%)
GERD (any)	88 (50%)
GERD(severe)	24 (14%)
GERD/PUD Complication (e)	31 (18%)
Bleeding	46 (26%)
Duration (yrs.)	
From ZES onset to the present study	
Mean ± SEM	13.7 ± 0.6
(Range)	(0.5–42.1)
From ZES onset to last follow-up/death (f)	
Mean ± SEM	19.4 ± 1.3
(Range)	(2.4–40.7)
From 1st NIH visit to last follow-up/death (f)	
Mean ± SEM	11.4 ± 0.9
(Range)	(1.2–23.7)

Abbreviations: ZES—Zollinger–Ellison syndrome; MEN1—Multiple Endocrine Neoplasia type 1; yrs.—years; NIH—National Institutes of Health; GERD—gastroesophageal reflux disease; PUD—peptic ulcer disease; and PMD—patient’s private medical doctor. (a) MEN1 was diagnosed using family history, serum calcium, prolactin, and PTH assays as described previously [100,156,157,158,159,160,161]. (b) Onset is defined as the onset of recurrent and/or persistent symptoms compatible with ZES as defined previously [106,162]. (c) Criteria for diagnosis of ZES required assessment of BAO/fasting gastrin/secretin test as described previously [104,150,163]. (d) Clinical symptoms were determined as described previously [164,165]. (e) Other GERD/PUD complications include nonbleeding complications (stricture [esophageal, duodenal, and small intestine], perforation, penetration, obstruction, and advanced Barrett’s esophagus) as defined previously [103,164,166]. (f) All patients were followed in the long term (≥5 years) at NIH and treated with gastric antisecretory drugs, except for patients who had an early death, surgical cure with low acid secretion [162,167,168,169], or returned to their PMD after diagnosis stabilization and evaluation [106]. During follow-up, 25 patients died, with 8 patients having a ZES-related death, and in 17 patients, the death was not ZES-related [101,170].

**Table 2:** Patient laboratory/tumor characteristics.

Characteristic	Number (%)
I. LABORATORY RESULTS	
BAO (mEq/h) (no gastric surgery) (a)	
Mean ± SEM	43.6 ± 2.0
(Range)	(1.8–159)
BAO (mEq/h) (previous gastric surgery) (a)	19.3 ± 2.6
Mean ± SEM	(8.2–33.1)
(Range)	
MAO (mEq/h) (no gastric surgery) (b)	
Mean ± SEM	66.7 ± 2.5
(Range)	(13–159)
MAO (mEq/h) (previous gastric surgery) (b)	
Mean ± SEM	28.4 ± 3.7
(Range)	(11.0–44.0)
Fasting serum gastrin (FSG) (pg/mL)	
Mean ± SEM	2717 ± 863
(Range)	(52–110,000)
Median	618
II. TUMORAL FEATURES	
Tumor extent (c)	
Overall tumor localization	
Localized disease	40 (23%)
Not localized	135 (77%)
Specific tumor extent (c)	
Primary only	68 (39%)
Primary and lymph node metastases	59 (34%)
Primary and liver metastases	39 (22%)
Primary tumor location (d)	
Duodenum	87 (50%)
Pancreas	30 (17%)
Lymph node primary (e)	22 (13%)
Other (f)	12 (12%)
Unknown (g)	36 (21%)

Abbreviations: BAO—Basal acid output; MAO—Maximal acid output; FSG—serum fasting gastrin. (a) 163 patients had a preoperative BAO (152 with no gastric surgery and 11 with previous gastric acid-reducing surgery) determined as described previously [104,150]. (b) A total of 143 patients had an MAO (133 with no gastric surgery and 10 with previous gastric acid-reducing surgery) determined as described previously [104,150]. (c) General tumor extent is determined by imaging and surgery in all patients as described previously [103,171,172,173]. Specific localization in 9 patients with regional disease could not be determined because no surgery was performed. Localized disease refers to patients with regional disease without distant metastases to liver/bone/or other sites. (d) The primary tumor site was established during surgery or endoscopy or by imaging as described previously [167,174,175,176,177,178]. (e) Primary lymph node gastrinomas were identified as described previously [179]. (f) Non-pancreatic-duodenal/lymph node primary sites occurred as described previously in the hepato-biliary tract [102,180], ovary, jejunum, mesentery, heart, lung cancer, and gastric antrum [103,181,182,183]. (g) Patients with diffuse liver metastases, with MEN1/ZES, or severe co-morbidities did not undergo routine surgical exploration as described previously [151,159,178,184], and the primary location, if not clearly identified on the imaging/endoscopy, was listed as an unknown primary site.

**Table 3:** Acid treatment data: surgical and medical (duration).

Characteristic	Number (% Total)
A. Gastric acid-reducing surgery	
I. Prior to the initial visit to the NIH	
No	11
Yes	164
II. Type of prior surgical treatment of acid hypersecretion	
Vagotomy-pyloroplasty/selective vagotomy	11 (4.6%)
Billroth I resection	4 (2.0%)
Billroth 2 resection	5 (3%)
III. Highly selective vagotomy at NIH (a)	22 (12.5%)
B. Medical treatment of acid hypersecretion prior to the present study (duration) (yrs.)	
I. Any medical acid treatment (yrs.) (*n* = 175) (b)	
Mean ± SEM	10.2 ± 0.5
Range	(0.1–30.1)
Any Tx > 10 yrs.	20 (11%)
II. Any treatment with H_2_R (yrs.) (*n* = 130) (b)	
Mean ± SEM	5.8 ± 0.4
Range	(0.2–20)
III. Any treatment with PPI (yrs.) (*n* = 169) (b)	
Mean ± SEM	5.6 ± 0.3
Range	(0–14.9)
PPI Tx > 10 yrs.	20 (11%)
IV. Time until medical acid treatment started (yrs.) (c)	
From ZES onset (yrs.) (c)	
Mean ± SEM	3.6 ± 0.4
(Range)	(0.0–26.0)
Prior to ZES diagnosis (yrs.) (*n* = 122) (c)	
Mean ± SEM	3.7 ± 0.4
(Range)	(0.01–26.2)
After ZES diagnosis (yrs.) (*n* = 51) (c)	
Mean ± SEM	1.5 ± 0.5
(Range)	(0.01–18.1)

Abbreviations: H_2_R—histamine H_2_-receptor antagonists; NIH—National Institutes of Health; PPI—proton pump inhibitors; ZES—Zollinger–Ellison syndrome; and others—see legends in Table 1 and Table 2. (a) Prior to the availability of PPIs, selective patients with high BAOs and antisecretory drug requirements, which can persist even after tumor resections, had a highly selective vagotomy performed at the time of any surgical exploration, as recommended from 1980 to 1983 [169,219,225]. (b) In the NIH perspective trials [106,214], H_2_Rs were the first effective acid antisecretory medical therapy, with cimetidine being first used in 1978, ranitidine in 1982, and famotidine in 1983 [198,226,227]. PPIs were first used in 1983 with omeprazole and with lansoprazole in 1989 [106,197,220,228]; so, all patients initially enrolled in this study were first treated with H_2_Rs (cimetidine, ranitidine, and famotidine) and later, most of them switched to PPIs (omeprazole and lansoprazole) while new patients generally started treatment with PPIs [106,229]. (c) The times of ZES onset and diagnosis were determined as described in the Methods section and Table 1 footnote. In 51 patients, the diagnosis of ZES was after or at the time of the earliest medical therapy, whereas in 122 patients, some initial medical therapy preceded the diagnosis of ZES, as defined in the Methods section.

**Table 4:** Acid treatment: drug schedule, and dose.

Characteristic	Number (% Total)
I. Treatment schedule	
I.A. First acid medical drug treatment (*n* = 175) (a)	
H_2_R	130 (74%)
PPI	45 (26%)
I.B. Total acid treatment: drug (*n* = 175) (a)	
H_2_R-related	
H_2_R antagonist (cimetidine, ranitidine, famotidine, and nizatidine) at any time	130 (74%)
Only H_2_R antagonists without any PPI treatment at any time	6 (3.4%)
H_2_R with an anticholinergic agent at any time (b)	16 (9.1%)
H_2_R without an anticholinergic agent at any time	158 (90%)
H_2_R followed by PPI (c)	124 (71%)
PPI-related	
PPI (omeprazole, lansoprazole, and pantoprazole) at any time	169 (96.5%)
PPI only	45 (26%)
PPI followed by H_2_R (d)	3 (1.7%)
II. Antisecretory drugs used at any time (a and e)	
II.A. H_2_R (a and e)	
Cimetidine	44 (25%)
Ranitidine	108 (62%)
Famotidine	18 (10%)
Nizatidine	3 (1.7%)
II.B. PPI (a and e)	
Omeprazole	168 (96%)
Lansoprazole	26 (15%)
Pantoprazole/esomeprazole	4 (2.2%)
III. Treatment: acid secretory drug dose	
III.A. Initial antisecretory treatment	
H_2_R (f)	
# of patients	128
Initial dose (mg/day)	
Mean ± SEM	918 ± 86
(Range)	(100–4800)
PPI	
# of patients	170
Initial dose (mg/day) (g)	
Mean ± SEM	71.4 ± 3.0
(Range)	(20–240)
III.B. Current study: antisecretory drug dose	
H_2_R	
# of patients	9
current dose (mg/day) (f)	
Mean ± SEM	1083 ± 391
(Range)	(300–3600)
PPI	
# of patients	166
Current dose (mg/day) (g)	
Mean ± SEM	61.7 ± 3.0
(Range)	(20–240)

Abbreviations; H2R—Histamine H2-receptor antagonists; PPI—Proton pump inhibitors; ZES—Zollinger–Ellison syndrome. (a) In the NIH perspective trials [106,214], H_2_Rs were the first effective acid antisecretory medical therapy, with cimetidine first used in 1978, ranitidine in 1982, and famotidine in 1983 [198,226,227]. PPIs were first used in 1983 with omeprazole and then with lansoprazole in 1989 [106,197,220,228]; so, all patients initially enrolled in this study were first treated with H_2_Rs (cimetidine, ranitidine, and famotidine) and later, most of them switched to PPIs (omeprazole and lansoprazole). New patients generally started treatment with PPIs [106,229]. (b) When only H_2_R antagonists were available, many patients required high and frequent dosing to control the acid hypersecretion [177,226,227]. The addition of an anticholinergic drug such as isopropamide or probanthine potentiated the H_2_R inhibitory effect and was thus frequently added [106,230]. (c) Patients with active disease were initially treated with H_2_Rs and then switched to PPIs as described previously [106]. (d) Patients with active disease were initially treated with PPIs and then switched to H_2_Rs, particularly after curative resections [169,225]. (e) The total number of patients treated with a given PPI or H_2_R in total was greater than the number of patients initially treated with PPIs/H_2_Rs because many of them received more than one antisecretory drug over time. (f) The daily H_2_R dosage is listed as a ranitidine-equivalent dose calculated as described previously [106,227] using their relative potencies of famotidine/ranitidine/cimetidine of 1:9:32 based on a previous study on ZES patients [227]. (g) The PPI dose listed is listed as a daily omeprazole-equivalent dose as described previously from data demonstrating that omeprazole (20 mg) was equivalent to 40 mg of esomeprazole, 30 mg of lansoprazole, 40 mg of pantoprazole, and 20 mg of rabeprazole [231].

**Table 5:** Mean VB_12_/MMA and plasma tHCY levels and % VB_12_-deficient patients according to different criteria.

Characteristic	Number (% Total)
A. Single serum measurements [VB_12_, MMA, and tHCY]	
I. VB_12_ levels	
Ia. VB_12_ level (pg/mL)	
Mean ± SEM	394 ± 14
(range)	(71–999)
Ib. Proposed VB_12_ deficiency	
# with VB_12_ level < 200 pg/mL (a)	18 (10%)
# with VB_12_ level 200–350 pg/mL (b)	67 (38%)
II. MMA levels	
IIa. MMA value (uM)	
Mean ± SEM	0.25 ± 0.01
(range)	(0.06–0.83)
IIb. Proposed VB_12_ deficiency < 200 pg/mL (c)	
# with MMA level > 0.26 (uM) (a)	53 (30%)
# with MMA level > 0.37 (uM) (b)	32 (18%)
IIII. Plasma tHCY levels	
IIIa. tHCY value (uM)	
Mean ± SEM	10.20 ± 0.41
(range)	(4.0–47)
IIIb. Proposed VB_12_ deficiency (d)	
# with tHCY level > 13 uM (a)	22 (13%)
# with tHCY level > 15 uM (a)	14 (8%)
B. Combination blood measurements [VB_12_, MMA, and tHCY]	
# with VB_12_ level < 200 pg./mL + MMA > 0.37 uM (e)	14 (8%)
# with VB_12_ level < 200 pg./mL + tHCY > 15 uM (e)	8 (4.6%)
# with MMA level > 0.37 uM or tHCY > 15 uM and response to VB_12_ administration [normal folate] (f)	37 (21%)

Abbreviations: MMA—serum methylmalonic acid level; tHCY—plasma total homocysteine level; and VB12 level: serum vitamin B_12_ concentration. (a) Numerous studies have proposed a serum VB_12_ level of <200 pg/mL for diagnosing VB_12_-deficient patients [112,113,114,115,118,120,233,235]. (b) Serum VB_12_ levels over the range of 200–350 pg/mL have been reported to represent a low-level range that could suggest VB_12_ deficiency [115,116,118]. (c) Many studies propose that an MMA level > 0.37 uM should be generally used to identify VB_12_-deficient patients [115,116,117,118] and a few recommend using an MMA level of >0.26 uM [116,235,236,237]. These recommendations are only for patients with normal renal function [113,116,236,238]. (d) Various studies have proposed either a tHCY level of >13 uM [113,115,234] or >15 uM [112,114,115,235] as the upper limit of normal. (e) Numerous combinations of serum VB_12_ levels with either MMA or tHCY levels have been reported to be more sensitive than either one alone [112,113,114,115,119,120]. (f) The response of blood MMA/tHCY with normal serum folate levels and renal function to the administration of crystalline VB_12_ either given orally or parenterally is considered by many to be one of the single best measurements of VB_12_ deficiency [112,114,117,236,237].

**Table 6:** Comparison of serum VB_12_/MMA and plasma tHCY levels in patients with or without VB_12_ deficiency.

	Number (% Total Group)		
	VB_12_ Deficiency		
	Yes	No	*p*-Value
Characteristic	(*n* = 37)	(*n* = 138)	
Serum VB_12_ levels (pg/ML)			
Mean ± SEM	254 ± 22	433 ± 15	<0.0001
(Range)	(71–665)	(150–999)	
# with VB_12_ level < 200 pg/mL (a)	16 (43%)	2 (1.4%)	<0.0001
# with VB_12_ level 200–350 pg/mL (b)	14 (38%)	53 (38%)	0.99
Serum MMA levels (uM)			
Mean ± SEM	0.46 ± 0.03	0.19 ± 0.01	<0.0001
(Range)	(0.14–0.83)	(0.06–0.42)	
# with MMA level > 0.26 (uM) (c)	31 (84%)	22(16%)	<0.0001
# with MMA level > 0.37 (uM) (c)	30 (81%)	2 (1.4%)	<0.0001
Plasma tHCY levels (uM)			
Mean ± SEM	15.3 ± 1.1	8.8 ± 0.3	<0.0001
(Range)	(7.0–41.0)	(4.0–47.0)	
# with tHCY level > 13 uM (d)	18 (49%)	4 (2.9%)	<0.0001
# with tHCY level > 15 uM (d)	14 (39%)	0 (0%)	<0.0001

Abbreviations: See legends in Table 1, Table 2, Table 3, Table 4 and Table 5. (a) Numerous studies have proposed a serum VB_12_ level of <200 pg/mL for diagnosing VB_12_-deficient patients [112,113,114,115,118,120,233,235]. (b) Serum VB_12_ levels over the range of 200–350 pg/mL have been reported to represent a low-level range that could suggest VB_12_ deficiency [115,116,118]. (c) Several studies propose classifying those with serum MMA > 0.37 uM as VB_12_-deficient patients [115,116,117,118] and a few recommend using a serum MMA level of >0.26 uM [116,235,236,237]. These recommendations are only for patients with normal renal function [113,116,236,238]. (d) Various studies have proposed either a plasma tHCY level of >13 uM [113,115,234] or >15 uM [112,114,115,235] as the upper limit of normal.

**Table 7:** Comparison of hematological/serum folate values in patients with or without VB_12_ deficiency.

	VB_12_ Deficiency		
	Yes	No	*p*-Value
Characteristic	(*n* = 37)	(*n* = 138)	
Hematological value (a)			
Hematocrit (%)	41.5 ± 0.6	40.9 ± 0.6	0.61
Mean corpuscular volume (fL)	90.8 ± 0.9	89.3 ± 0.9	0.70
Leukocytes (×10^3^/mm^3^)	6.1 ± 0.3	6.4 ± 0.1	0.32
Red blood cell count (×10^3^/mm^3^)	4.6 ± 0.9	4.7 ± 0.4	0.67
Serum folate levels (ng/mL)			
Mean ± SEM	11.6 ± 0.77	11.4 ± 0.43	0.70
Median (range)	10.60 (4.4–24.5)	10.50 (3.8–30.0)	

(a) All hematological/folate values are from the time of the admission, with the blood MMA, tHCY, and VB_12_ levels shown in Table 5, Table 6, Table 7 and Table 8, and were performed by the NIH Clinical Chemistry Laboratories.

**Table 8:** Comparison of ZES Clinical/lab/tumoral features in patients with or without VB_12_ deficiency.

	Number (% Total Group)		
	VB_12_ Deficiency		
	Yes	No	*p*-Value
Characteristic	(*n* = 37)	(*n* = 138)	
I. Clinical features/disease course			
Age at study (yrs.) (Mean ± SEM) (a)	54.1 ± 2.0	53.7 ± 1.0	0.93
Male gender	24 (65%)	67 (49%)	0.096
Race			
White	30 (81%)	107 (78%)	0.82
Nonwhite	7 (19%)	31 (23%)	
Age at ZES onset (yrs.) (Mean ± SEM) (a)	40.3 ± 2.0	39.8 ± 1.0	0.71
Presenting symptom (a)			
Pain	29 (78%)	106 (77%)	0.99
GERD	15 (40%)	72 (52%)	0.27
Diarrhea	32(86%)	108 (78%)	0.36
MEN-1 present (a)	9 (24%)	36 (26%)	0.84
Duration (yrs.) (mean ± SEM)			
Time ZES onset to study	13.8 ± 1.3	13.7 ± 0.7	0.93
II. LABORATORY RESULTS			
BAO (mEq/h) (Mean ± SEM) (b)	41.7 ± 3.8	42.2 ± 2.2	0.82
MAO (mEq/h) (Mean ± SEM) (c)	57.3 ± 4.8	66.2 ± 2.9	0.10
# Previous gastric acid-reducing surgery) (a)	2 (5.4%)	9 (6.5%)	0.99
Fasting serum gastrin (FSG) (pg/mL))			
Mean ± SEM	1953 ± 401	2921 ± 1090	0.84
(Range)	(172–8900)	(52–110,000)	
Median	742	597	
III. TUMORAL FEATURES			
Tumor extent			
Overall tumor localization (d and e)			
Localized disease	29 (78%)	106 (77%)	0.99
Not localized	8 (22%)	32 (23%)	
Specific tumor extent (e and f)			
Primary only	10 (27%)	58 (42%)	0.13
Primary and lymph node metastases	17 (46%)	42 (30%)	0.082
Primary and liver metastases	8 (22%)	31 (22%)	0.99
Not established: no surgery	2 (5.4%)	7 (5.1%)	0.99
Primary tumor location (f)			
Duodenum	21 (55%)	66 (48%)	0.47
Pancreas	8 (22%)	22 (16%)	0.46
Lymph node primary (g)	5 (14%)	17 (12%)	0.78
Other (h)	2 (5%)	10 (7%)	0.99
Unknown (i)	6 (16%)	30 22%)	0.65

Abbreviations: BAO-Basal acid output; MAO- Maximal acid output; FSG-serum fasting gastrin. (a) See legends in Table 1, Table 2, Table 3, Table 4, Table 5 and Table 6 for an explanation of variables. (b) A total of 163 patients had a preoperative BAO (152 with no gastric surgery and 11 with previous gastric acid-reducing surgery) determined as described previously [104,150]. (c) A total of 143 patients had an MAO (133 with no gastric surgery and 10 with previous gastric acid-reducing surgery) determined as described previously [104,150]. (d) Localized disease included patients with regional disease without distant metastases as defined previously [171]. (e) General tumor extent determined by imaging and surgery in all patients as described previously [103,171,172]. Specific localization in 9 patients with regional disease could not be determined because no surgery was performed. (f) The primary tumor site was established during surgery or endoscopy or by imaging as described previously [167,174,175,177]. (g) Primary lymph node gastrinomas were identified as described previously [103]. (h) Non-pancreatic-duodenal/lymph node primary sites occurred, as described previously, in the hepato-biliary tract [102,180], ovary, jejunum, mesentery, heart, lung cancer, and gastric antrum [103,181,182,183]. (i) Patients with diffuse liver metastases, with MEN1/ZES, or severe co-morbidities did not undergo routine surgical exploration, as described previously [151,159,178], and the primary location, if not clearly identified on the imaging/endoscopy, was listed as the primary site unknown.

**Table 9:** Comparison of antisecretory treatment results in patients with or without VB_12_ deficiency.

	Number (% Total Group)		
	VB_12_ Deficiency		*p*-Value
	Yes	No	
Characteristic	(*n* = 37)	(*n* = 138)	
I. Type antisecretory treatment			
# Previous gastric acid-reducing surgery (a)	2 (5.4%)	9 (6.5%)	0.99
Medical treatment:	37 (100%)	138 (100%)	
At the time of the present study			
H_2_R (*n* = 9)	0 (0%)	9 (7%)	0.21
PPI (*n* = 166)	37 (100%)	129 (93%)	
During 10 yrs. prior to the present study			
H_2_R only (*n* = 6)	0 (0%)	6 (4.3%)	0.34
PPI only (*n* = 150)	34 (92%)	116 (84%)	0.72
Any PPI (*n* = 166)	37 (100%	129 (93%)	0.21
H_2_R 1st then PPI (*n* = 17) (b)	3(8.1%)	15 (11%)	0.99
PPI > 5 yrs. (*n* = 93)	25 (68%)	68 (49%)	0.063
II. General medical/surgical acid treatment features			
Age at 1st medical treatment (yrs.)	43.9 ± 2.0	43.6 ± 1.0	0.88
Age at H_2_R initial treatment (yrs.)	41.7 ± 2.6	43.2 ± 1.0	0.54
Age at PPI initial treatment (yrs.)	48.0 ± 2.0	47.6 ± 1.0	0.88
III. Duration of antisecretory Tx (yrs.) (mean ± SEM)			
ZES onset to any acid Tx (*n* = 175) (a)	3.8 ± 0.9	3.7 ± 0.4	0.92
ZES onset to PPI started (*n* = 167) (a)	7.6 ± 1.2	7.9 ± 0.6	0.76
Initial acid treatment to the present study (*n* = 175)	10.2 ± 1.0	10.1 ± 0.6	0.76
Initial H_2_R treatment to PPI Tx (Tx PPI/H_2_R) (*n* = 124)	6.0 ± 1.1	5.9 ± 0.5	0.96
PPI Tx (+/− with H_2_R) prior to present study (all PPI) (*n* = 169)	6.2 ± 0.6	5.5 ± 0.3	0.22
Time treated only with H_2_R prior to present study (*n* = 6)	0	15.0 ± 1.6	0.0022
Time treated only with PPI prior to the present study (*n* = 45)	3.2 ± 0.6	3.4 ± 0.5	0.73
IV. Gastric acid control and VB_12_ status			
IV.A. Correlations with present study for single admission results (*n* = 175)			
Control acid output (mEq/h) (c)			
mean ± SEM	0.14 ± 0.04	1.71 ± 0.17	<0.0001
(range)	(0–1.10)	(0–9.5)	
Control acid pH			
mean ± SEM	6.4 ± 0.0.2	3.7 ± 0.2	<0.0001
(range)	(4.0–7.6)	(1.2–7.5)	
Number with control acid with pH < 3.5 (d)	0 (0%)	78 (56%)	<0.0001
Number with control acid with pH ≥ 7	18(49%)	12 (8.5%)	<0.0001
IV.B. Correlation with acid control results for all admissions over previous 5 yrs. (*n* = 873)			
With sustained achlorhydria (>50% Adm acid = 0) (e)	27 (73%)	33 (24%)	<0.0001
With sustained hypochlorhydria (0.1 to <1 mEq/h (>50%) (e)	10(27%)	37(27%)	0.99
With >50% acid controls ≥1 mEq/h (e)	0 (0%)	68 (49%)	0.0005

Abbreviations: Adm—admission; BAO—Basal acid output; MAO—Maximal acid output; FSG—serum fasting gastrin; and Tx—treatment. For other abbreviations, see legends in Table 1, Table 2, Table 3, Table 4, Table 5, Table 6, Table 7 and Table 8. (a) See legends in Table 1 and Table 2 for an explanation of each of these characteristics. (b) In the NIH perspective trials [106,214], H_2_Rs were the first effective acid antisecretory medical therapy, with cimetidine first used in 1978, ranitidine in 1982, and famotidine in 1983 [198,226,227]. PPIs were first used in 1983 with omeprazole and then with lansoprazole in 1989 [106,197,220,228]; so, all patients initially enrolled in this study were first treated with H_2_Rs (cimetidine, ranitidine, and famotidine) and later, most of them switched to PPIs (omeprazole and lansoprazole), while new patients generally started treatment with PPIs [106,229]. (c) All antisecretory drug doses were determined as described previously based on the results of the acid control secretory rate [104,106,164,227]. This was determined by assessing the drug acid control secretory rate for the hour prior to the next antisecretory dose, and the antisecretory dose was adjusted to control the acid hypersecretion to <10 mEq/h in the majority of patients [104,106,164,227] or to below <5 mEq/h in patients with moderate/severe GERD [103,166] or previous Billroth resections depending on UGI endoscopic findings and symptom control [166,222]. These levels have been shown to result in the healing of mucosal lesions and, if maintained, prevent the development of additional peptic mucosal damage [164,177,222,226]. (d) The frequency of gastric acid > pH 3.5 was included because studies reported that pepsin activation, which cleaves food-bound VB_12_ during digestion and is essential for VB_12_ absorption, is inhibited at pH levels > 3.5 [79,243]. (e) Using the 873 gastric acid drug control analysis performed over the 5 years of this study (19967–2001), the patients were divided into one of three acid control categories: the presence of sustained achlorhydria (>50% admission acid control = 0), sustained hypochlorhydria (acid control levels from 0.1 to <1 mEq/h > 50%), and >50% acid controls ≥1 mEq/h. This categorization was determined as described in the Methods section and previous studies [54,106,244].

## Data Availability

Data is contained within the article.
